# Non-isothermal TGA, mechanism and thermodynamic study of the thermal degradation of mixed metal Zr/W-UiO-66-type MOF materials

**DOI:** 10.1039/d6ra04868d

**Published:** 2026-10-03

**Authors:** Cyrille Ghislain Fotsop, Alexandra Lieb, Franziska Scheffler

**Affiliations:** a Otto-von-Guericke-University Magdeburg, Chemical Institute, Chair for Industrial Chemistry Universitätsplatz 2 39106 Magdeburg Germany cyrille.fotsop@ovgu.de franziska.scheffler@ovgu.de

## Abstract

The thermal stability and decomposition behavior of Zr-based UiO-66 metal–organic frameworks (MOFs) are crucial for their use in practical thermocatalytic and high-temperature applications. This study systematically investigated the thermokinetic and thermodynamic behavior of pristine UiO-66 and tungsten-modified Zr/W-UiO-66 MOFs containing 25% and 60% W to elucidate the effect of W incorporation on their thermal decomposition pathways. The activation energy and pre-exponential factor were determined using model-free Flynn–Wall–Ozawa (FWO) and Kissinger–Akahira–Sunose (KAS) methods, combined with an iterative procedure. Thermogravimetric analysis revealed distinct multistep decomposition pathways, with total mass losses of 50.5% for UiO-66, and 35.3% and 35.2% for Zr/W-UiO-66 (25% and 60%, respectively). The apparent activation energies ranged from 80.5 to 363.8 kJ mol^−1^ depending on the W content and decomposition stage. This demonstrates the strong influence of tungsten incorporation on the thermal decomposition energy landscape. Mechanistic analysis identified random nucleation and subsequent growth (G5), three-dimensional diffusion (*D*_8_) and three-dimensional diffusion under spherical symmetry (*D*_3_) as the most probable mechanisms for successive decomposition steps. The thermodynamic parameters (Δ*S*^≠^, Δ*H*^≠^ and Δ*G*^≠^) obtained from the FWO and KAS approaches were highly consistent, with deviations of approximately 6%. Furthermore, PXRD, SEM-EDX, FTIR and N_2_ sorption analyses confirmed the structural and textural evolution of the materials, providing evidence of tungsten's role in the Zr/W-UiO-66 framework. Combined thermokinetic and structural analyses provide new insights into the relationships between W incorporation, decomposition mechanisms and the formation of ZrO_2_ and ZrO_2_@WO_3_-derived hybrid oxides. These findings establish a comprehensive framework for understanding and controlling the thermal transformation of W-modified UiO-66 MOFs toward functional oxide materials.

## Abstract 2

The thermal stability and decomposition behavior of Zr-based UiO-66 metal–organic frameworks (MOFs) are crucial for their use in practical thermocatalytic and high-temperature applications.

## Graphical abstract

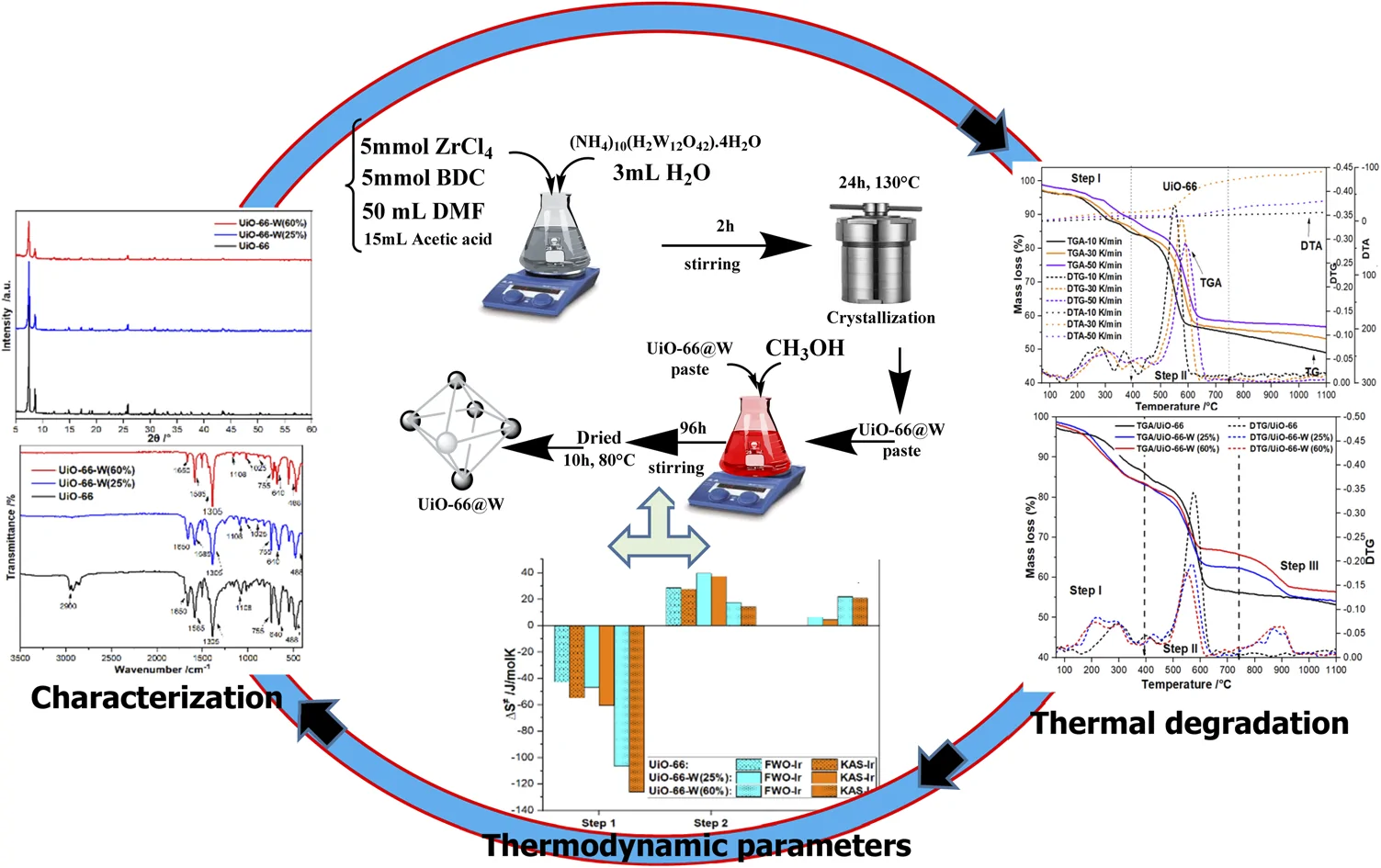

## 1 Introduction

The Zr-based metal–organic framework (MOFs) are widely studied compounds due to their high thermal, mechanical and chemical stability. UiO-66 consists of zirconium oxide (Zr_6_O_4_(OH)_4_) clusters linked to the 1,4-benzodicarboxylic acid (BDC) ligand, so that the whole structure forms a 3D porous network with octahedral (effective diameters: *d* = 11 Å) and tetrahedral (8 Å) cages interconnected to form a pore size of ∼6 Å. UiO-66 is known in the literature for its stable catalytic properties,^1,2^ its ability for the post-synthetic modification,^3,4^ its high thermal stability^5,6^ and its wide range of applications in adsorption^7^ and separation.^8,9^ This is one of the most extensively studied Zr-based MOFs. Shearer *et al.* 2014, reported on the fine tuning of defect removal in the UiO-66 structure, on which the impact of different synthesis conditions, the physicochemical properties of UiO-66 were further investigated, for this purpose the influence of the BDC/Zr ratio and the synthesis temperature were evaluated, they found that the synthesis of UiO-66 was ideal when the synthesis conditions were moderate. They found that increasing the BDC/Zr ratio (2 : 1) and the synthesis temperature (220 °C) shifted the equilibrium of the synthesis solution in preference to BDC-Zr bonds, which would lead to BDC-linker deficiencies.^10^ Shearer *et al.* 2013 reported on the *in situ* characterization study using infrared spectroscopy and gravimetry during the dehydroxylation and solvent removal in UiO-66 and UiO-67; for this purpose, DRIFTS and TG-DSC analyses were performed *in situ* during dehydration, dehydroxylation and rehydration.^11^ The spectra obtained showed several bands due to isolated OH groups, and the OH vibrational frequencies of fully hydrated, partially dehydrated and fully dehydrated Zr_6_-node clusters were studied on the basis of the different geometric configurations of the Zr_6_-node clusters; rehydroxylation showed the presence of new vibrational bands in both materials. Theoretical studies have allowed us to propose a possible explanation for the behavior of the samples and to calculate the dehydration energies.^11^ Cliffe *et al.* 2014 further reported that the use of formic acid as a modulator favored the achievement of UiO-66, with missing cluster defects, which were visible in correlated nanoregions with a reo topology and reduced linker connectivity (8) comparable to the UiO-66 ideal (12) as missing linker defects.^12^ Shearer *et al.* 2016 evaluated the defect chemistry in UiO-66 synthesized using different monocarboxylic acids as modulators according to the current synthesis conditions. To achieve this, several systematic studies were evaluated during the synthesis of UiO-66, in which only the acidity (p*K*_a_) and/or the concentration of the modulator was evaluated; they found that, the composition and porosity of the final material was influenced by the concentration of the monocarboxylic acids. TGA, ATR-IR, PXRD and NMR analyses were used to prove the presence of the defects. They found that linker defects and missing clusters were observed depending on the nature of the modulating acid used, and that the concentration of linker cluster defects increased with the concentration and acidity of the modulator. However, the existing defects could be replaced by a mixture of formate molecules derived from the decomposition of the synthesis solvent and deprotonated modulators.^5^

The above results suggest that kinetic studies and the evaluation of activation energies for the decomposition of UiO-66 remain unexplored. However, some important research questions on the nature of the products derived from the thermal decomposition of UiO-66 remain to be explored. The literature suggests that when UiO-66 is heated above 300 °C to 500 °C, the Zr_6_O_4_(OH)_4_ clusters,^13,14^ undergo a reversible structural change to Zr_6_O_6_ leading to the formation of ZrO_2_ by dehydroxylation and recombination of the Zr_6_-node clusters.^14^ The thermal decomposition of UiO-66 is affected by defects in the Zr_6_ clusters and BDC linkers. These defects can impact pore accessibility, mass transfer and the release of volatile decomposition products. The incorporation of polyoxotungstate species can modify these structural and diffusional characteristics further by partially occupying accessible pore regions and altering the local chemical environment of the Zr_6_ clusters and linkers. Consequently, W incorporation can modify the kinetic and thermodynamic barriers associated with the different decomposition stages. The aim of this study is therefore to establish a relationship between W loading, pore accessibility, mass transfer and the thermal decomposition kinetics of UiO-66. The incorporation of W into UiO-66 is not just to modify the framework composition; it is also to investigate the effect of W incorporation on the thermal decomposition behavior, kinetic parameters and thermodynamic characteristics of the Zr-based MOF. UiO-66 contains Zr_6_O_4_(OH)_4_ clusters which dehydrate and dehydroxylate upon heating, eventually forming ZrO_2_. The introduction of W into the UiO-66 structure provides an additional metal component that can modify the thermal stability and decomposition pathway of the Zr-based framework. This may also promote the formation of mixed Zr–W oxide phases, such as ZrO_2_/WO_3_ hybrid materials, following thermal treatment. However, thermokinetic and thermodynamic studies of the dehydration and dehydroxylation properties of UiO-66 remain poorly understood. This requires a thorough understanding in the context of the kinetics and thermodynamics of pyrolysis. This has led us to carry out a series of systematic studies not only on UiO-66, but also on its tungsten-doped forms, including thermodynamic studies and the influence of the heating rate during the thermal decomposition according to the hypothesis of the formation of hybrid oxides (ZrO_2_@WO_3_), as a catalyst for photocatalytic applications.^15,16^ To ensure the relevance of our research, all the synthesis reactions were carried out under the same conditions. Two concentrations of tungsten (*W*), *i.e.* 25% and 60%, were incorporated into the UiO-66 cavities by *in situ* treatment. Various characterizations by PXRD, SEM, EDX, nitrogen adsorption and FT-IR spectroscopy were carried out on the samples in order to evaluate the effect of the tungsten concentration on the properties of UiO-66. Our results were also validated by comparing the experimental data for zirconia and hybrid oxides with those reported in the literature.

The aim of this work is to investigate the thermal decomposition of UiO-66 and Zr/W-UiO-66 MOFs and identify its by-products (ZrO_2_ and hybrid oxide) using isoconversion methods. Prior to the thermal composition study, the synthesis of zirconium MOFs was carried out. The kinetic parameters of pyrolysis, such as the activation energy (*E*_a_) and the pre-exponential factor (*A*), were evaluated using model-free methods, such as the Flynn–Wall–Ozawa (FWO) and Kissinger–Akahira–Sunrose (KAS) models, and following an iterative procedure for the FWO-Ir and KAS-Ir models.^17,18^ Subsequently, the most probable mechanism function controlling the thermal decomposition of Zr/W-UiO-66 was then determined using the multiple heating rate (MHR) method and the thermodynamic parameters Δ*S*^≠^, Δ*H*^≠^ and Δ*G*^≠^.

## 2 Material and experimental procedure

### 2.1 Materials

Zirconium(iv) chloride (ZrCl_4_) (99.9%), benzene-1,4-dicarboxylic acid (BDC) (99.9%), ammonium metatungstate hydrate ((NH_4_)_6_H_2_W_12_O_40_*x*H_2_O)) (99.5%), methanol (99.5%), *N*,*N*-dimethylformamide (DMF) (99.8%), acetic acid (99%), methanol (99.5%), all from technical grade, Sigma-Aldrich, St. Louis, USA) and distilled water were used without further purification during the synthesis of Zr/W-UiO-66.

### 2.2 Synthesis of UiO-66 and Zr/W-UiO-66

For the synthesis of UiO-66, 5 mmol ZrCl_4_ was dissolved in 30 mL DMF and 5 mmol BDC in 35 mL DMF separately for 40 min, the two solutions were mixed, followed by the addition of 3 mmol acetic acid. The final mixture was stirred for 2 h at room temperature and then transferred to a Teflon-lined autoclave for recrystallization at 120 °C for 24 h. After cooling, the product obtained was washed 3 times with DMF then stirred in 50 mL methanol for 24 h to exchange the solvent, the final mixture was centrifuged and washed twice with methanol then oven dried at 65 °C overnight, the final product obtained was graded as UiO-66. The synthesis of the different Zr/W-UiO-66 was carried out using two concentrations of tungsten (W), *i.e.* 25 wt% and 60 wt%. The tungsten was incorporated *in situ* during the reaction by a one-step solvothermal method, following the same procedure as above, but 25 wt% and 60 wt% of (NH_4_)_6_H_2_W_12_O_40_*x*H_2_O) were dissolved in 5 mL of water and then added to the solution mixture of ZrCl_4_, BDC followed by the addition of 3 mmol of acetic acid. After washing and drying, the final product was recorded as UiO-66-W (25%) and UiO-66-W (60%). The experimental procedure is shown in Scheme 1.

**Scheme 1:**
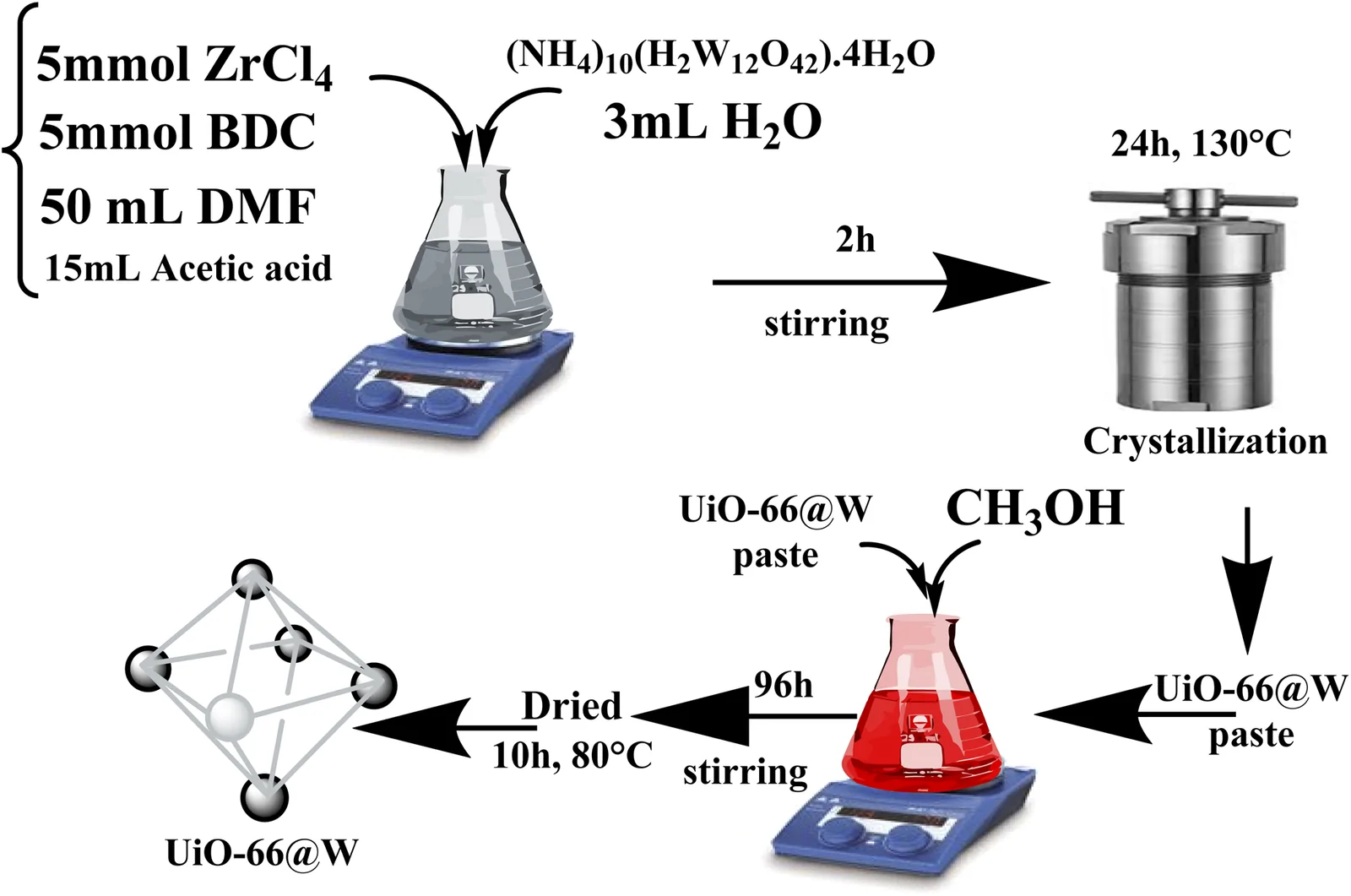
Synthesis of UiO-66 and Zr/W-UiO-66.

### 2.3 Experimental procedure

#### 2.3.1 Thermal decomposition

The thermal decomposition of UiO-66 and Zr/W-UiO-66 may be affected by factors such as the surrounding atmosphere, heating rate, moisture and temperature programme. In this study, thermogravimetric analysis experiments were performed under identical and controlled conditions to ensure a reliable comparison could be made between pristine and W-modified metal–organic frameworks (MOFs). As these parameters may affect the decomposition pathways and apparent kinetic parameters, the reported thermokinetic values should be interpreted in the context of the specific experimental conditions employed. This study focuses on the effect of tungsten incorporation on the thermal decomposition behavior and mechanisms of UiO-66, rather than on systematically investigating individual environmental parameters. Thermal decomposition of UiO-66 and Zr/W-UiO-66 was carried out in a muffle furnace. Thermogravimetric analysis was carried out at heating rates of *β* = 10, 30 and 50 K min^−1^ over a temperature range of 25–1100 °C under an inert argon atmosphere. Approximately 30 mg of each sample was used. The UiO-66 and Zr/W-UiO-66 samples treated as a function of each heating rate were designated UiO-66-(*x*), UiO-66-W (25%)-(*x*) and UiO-66-W (60%)-(*x*) where *x* is the heating rate. The derived curves were analyzed using Origin™ 2018 software. Thermodynamic and thermokinetic parameters were evaluated using the FOW, KAS, FOW-Ir and KAS-Ir equations using the Microsoft Excel solver.

### 2.4 Characterization techniques

The identity of all samples was determined by XRD using an Empyrean diffractometer (PANanalytical, Almelo, The Netherlands) equipped with a Cu-tube (*λ*: K_α1_ = 1.540 598 Å and K_α2_ = 1.544 426 Å) operating at 40 mA and 40 kV. All samples were scanned from 4–90° 2*θ*. The morphology and microstructure of all samples was recorded with an SEM XL30 FE SEM (FEI, Hillsboro, USA) at an acceleration voltage of 20 kV. FT-IR data was recorded using a Nicolet iS50 IR spectrometer (Thermo Scientific, Schwerte, Germany) in the 4000–200 cm^−1^ wavenumber range. TGA and DTA were performed using an STA 449C Jupiter (Netzsch, Selb, Germany) thermogravimetric analyzer. N_2_ sorption isotherms were recorded using a Micromeritics 3Flex instrument (Micromeritics, Norcross, USA). For the sorption analysis, all samples were activated to 250 °C under vacuum.

### 2.5 Theoretical approach

The thermal decomposition of UiO-66 and Zr/W-UiO-66 can generally be considered as a one-step solid state reaction (eqn (1)).1
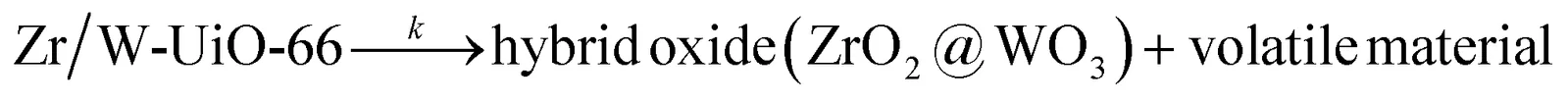
Here, *k* is the rate constant (eqn (2)) and “volatile material” the solvent during the thermal treatment.2
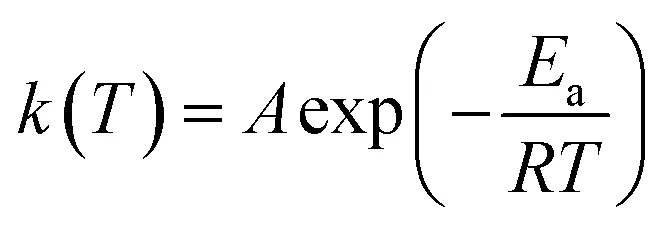


*E*_a_ is the activation energy (kJ mol^−1^), *A* the pre-exponential factor (min^−1^), *T* the absolute temperature (K) and *R* the gas constant (J K^−1^ mol^−1^).

Non-isothermal kinetic analysis is a model-free method, in which measurements of the corresponding temperatures *T* are functions of the degree of conversion *α* (eqn (3)) and a fixed value at different heating rates *β* (eqn (4)).3
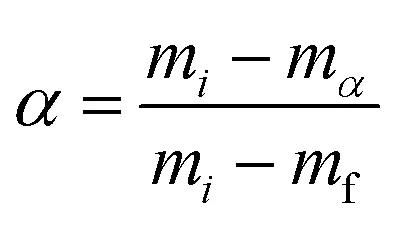
4
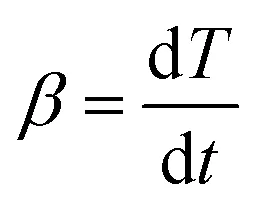
Here *m*_*i*_ is the initial mass of Zr/W-UiO-66, *t* is the time taken (min), *β* is the heating rate (K min^−1^), *m*_*α*_ is the current mass at a given degree of conversion *α* and *m*_f_ is the final mass.

Based on the kinetic theory, the kinetic equation of the thermal decomposition of solid materials is eqn (5).^19,20^5
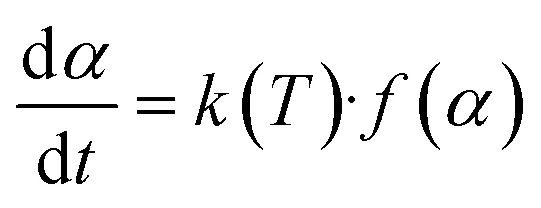
where *f*(*α*) is the reaction mechanism function.

Eqn (5) can be modified to eqn (6).6
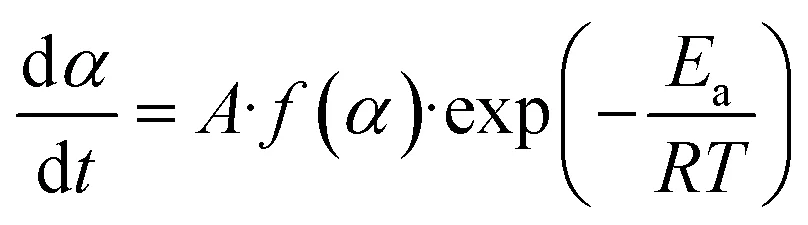


The substitution of eqn (4) within eqn (6) after adjustment, gives eqn (7), which is the fraction of material converted in a specified time.7
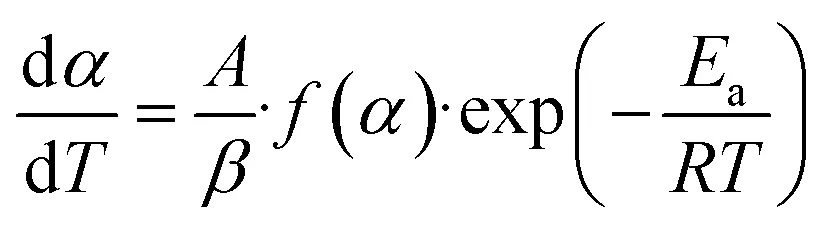


Eqn (7) can be solved by several methods. The integral form solution depends on the expression of the explicit function *f*(*α*) (Table S1)^17,21^ and its mechanism. The kinetic parameters of a solid state reaction are calculated from the heating rates and linear fitting of TGA and DTG data as a function of the *α* and *T*.^17,22^ The transposition and integration of eqn (7) gives eqn (8).^23^8
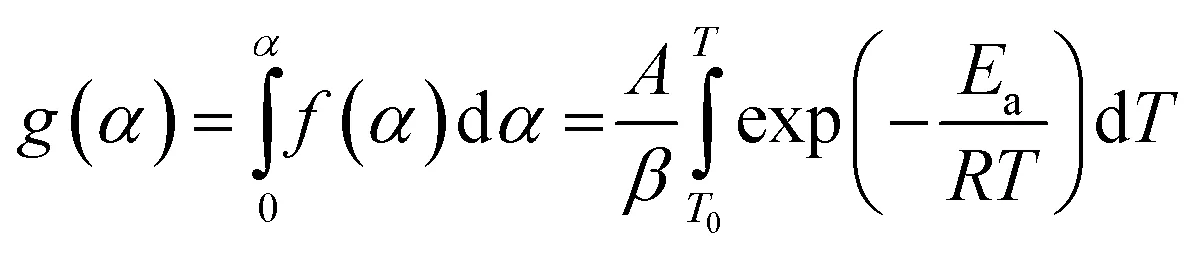
Here *g*(*α*) represents the integral form corresponding to each differential form of the function *f*(*α*). *T*_0_ and *T* are the initial and final temperatures for the reaction.

#### 2.5.1 Calculation of the activation energy *E*_a_

*E*_a_ was determined using integral methods based on KAS and FWO models,^24^ which consider DTG curves and correctly describe the calculation of kinetic parameters. From the integral methods, the conversion is influenced by *T* and *β*.^24,25^

The integral methods of FWO shown in eqn (9) (ref. 26 and 27) and KAS given by eqn (10) (ref. 29) are as follows:9
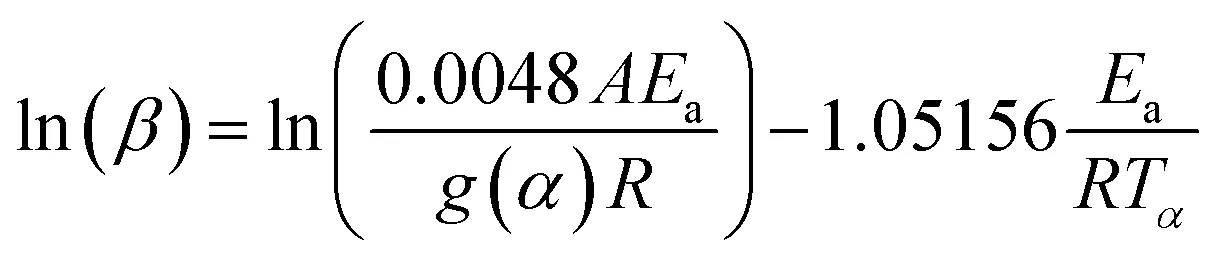
10
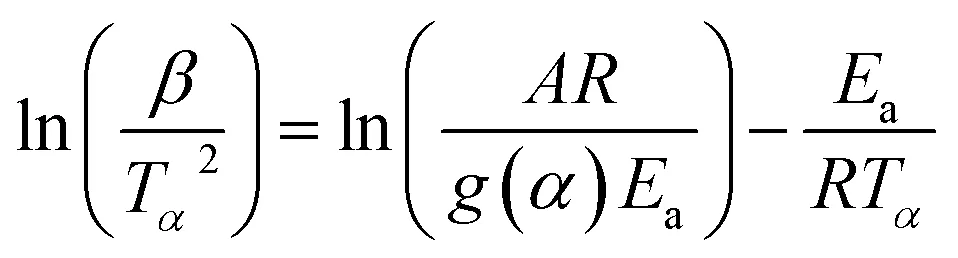


The *E*_a_ and *A* are obtained from the slope and intercept of the linear part of the plot of ln(*β*) (FWO) and ln(*β*/*T*_*α*_^2^) (KAS) *versus* 1000/*T*_*α*_, according to eqn (9) and (10), respectively.^28^

The iterative procedure^17,21^ was used to determine the most accurate value of *E*_a_ using the FWO-Ir (eqn (11)) and KAS-Ir (eqn (12)) methods.11
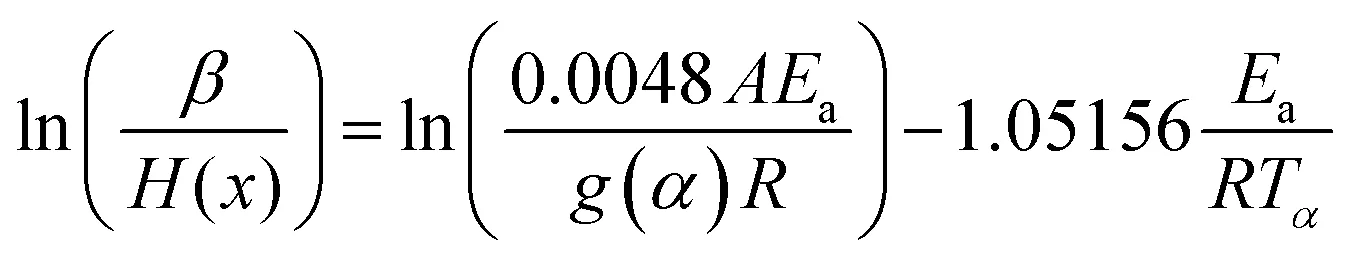
12
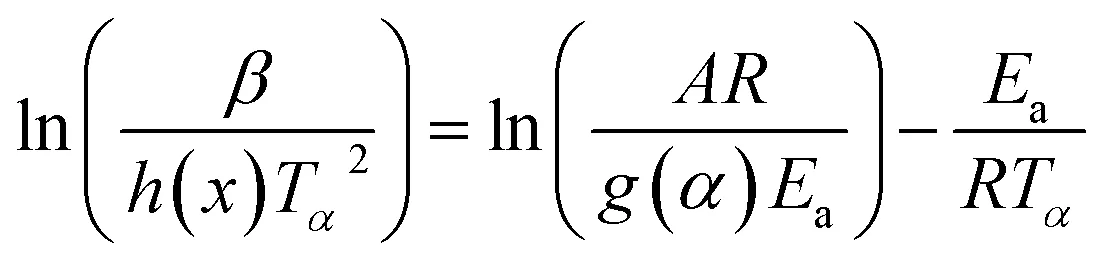
Here *h*(*x*) represents the 4th Senum and Yang approximation formula eqn (13) (ref. 30) and *H*(*x*) is shown in eqn (14).13
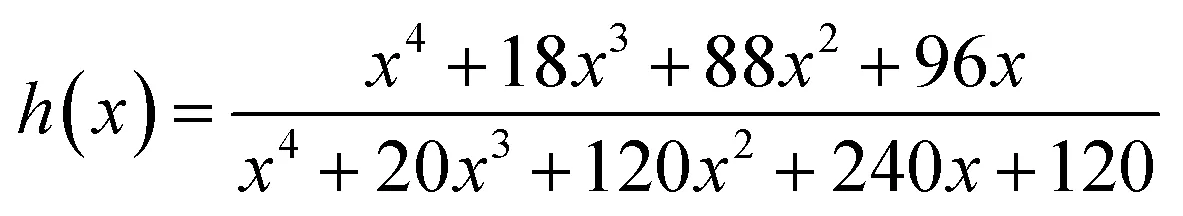
where *x* = *E*_a_/R*T*.14
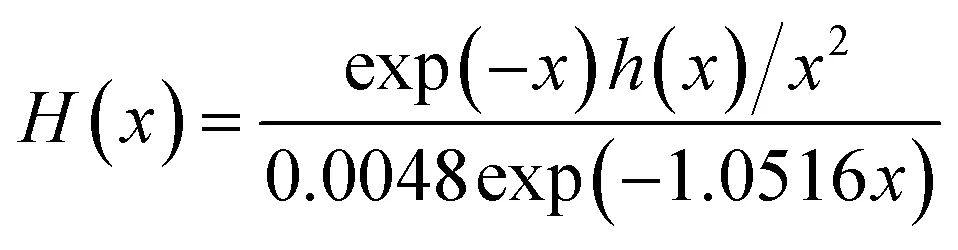


The iterative procedure^17,31^ followed these steps:

(i) It was assumed that *h*(*x*) or *H*(*x*) = 1. Then the initial value of *E*_a_ (*E*_a1_) was calculated using FWO eqn (9) and KAS eqn (10). The model-free methods stop the calculation at this step.

(ii) The value of *E*_a1_ was used to calculate the value *E*_a2_ from the plot of ln[*β*/*H*(*x*)] (FWO-Ir) or ln[*β*/*h*(*x*)*T*_*α*_2] (KAS-Ir) *versus* 1000/*T*_*α*_ using eqn (11) and (12).

(iii) Step (ii) was then repeated after replacing of *E*_a1_ with *E*_a2_. This procedure was repeated until *E*_ai_–*E*_a(i−1)_ < 0.01 kJ mol^−1^. Thus, the last value of *E*_ai_ was considered as the most accurate value of *E*_a_ of the reaction.

#### 2.5.2 Determination of the most probable mechanism function *g*(*α*)

The FWO and KAS models do not include *g*(*α*). Therefore, it was determined using the multiple heating rate (MHR) method.^32^ The MHR method was used in its linear form, derived by integrating of the right-hand side of eqn (8) which gives in eqn (15) with *x* = *E*_a_/*RT*.^33^15
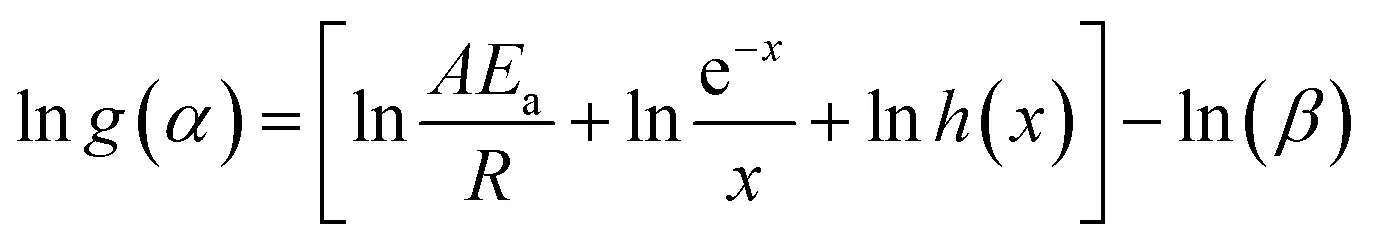


Linear regression of the plot of ln *g*(*α*) *versus* ln(*β*) provides an information on the probability that *g*(*α*) represents the reaction mechanism. The closer the slope is to −1 and the closer the linear correlation coefficient *R*^2^ is to 1, the more likely it is that the tested function describes the reaction mechanism. Eqn (15) shows that *A* and *E*_a_ have no effect on *g*(*α*). Thirty-six functions (Table S1) were tested with the MHR method to obtain the most likely probable function, where different values of conversion *α* corresponding to each heating rate *β* taken at the same temperature value were implemented in eqn (15).^18,21^

#### 2.5.3 Calculation of the pre-exponential factor *A*

*A* was determined using the intercepts of the plots of the FWO-Ir and KAS-Ir models (eqn (11) and (12)), after inserting the values of *E*_a_ corresponding to each conversion rate *α*, the gas constant *R* and the most probable mechanism function *g*(*α*).^34^

#### 2.5.4 Determination of the thermodynamic parameters Δ*S*^≠^, Δ*H*^≠^ and Δ*G*^≠^ of the thermal decomposition of UiO-66 and Zr/W-UiO-66

The relationship between the thermodynamic parameters of the conversion process and the kinetic parameters is a function of the correlation between the Zenera and Wertera or Eyring and Arrhenius laws, related to the reaction rate constant *k*(*T*).^35,36^ Δ*S*^≠^, Δ*H*^≠^ and Δ*G*^≠^ were calculated using eqn (16)–(18), respectively.16
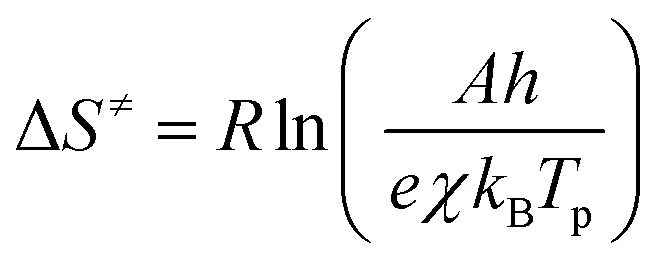

17 Δ*H*^≠^ = *E*_*α*_ − *RT*_P_

18 Δ*G*^≠^ = Δ*H*^≠^ − *T*_P_Δ*S*^≠^

Herein *E*_*α*_ is obtained from the iterative procedure using the FWO-Ir and KAS-Ir models, *T*_p_ is the peak temperature of the DTG curve, *A* is the pre-exponential factor, *h* is Planck's constant (*h* = 6.6269 × 10^−34^ Js), *e* is the Neper number (*e* = 2.7183), *χ* is the transition factor equal to unity,^19,37^*k*_B_ is the Boltzmann constant (*k*_B_ = 1.3819 × 10^−23^ J K^−1^) and *R* is the gas constant (*R* = 8.314 J mol^−1^ K^−1^).

## 3 Results and discussion

### 3.1 XRD analysis of UiO-66, Zr/W-UiO-66 and their thermal decomposition products

The PXRD pattern of UiO-66, UiO-66-W (25%), UiO-66-W (60%) and its by-products ZrO_2_ and hybrid oxide are shown in Fig. 1(a and b). The formation of the UiO-66 material is observed by standard peaks for 2*θ* between 5 and 10, indicating that the Zr_6_ composed of zirconium clusters Zr_6_O_4_(OH)_4_ is released from the BDC linker to form a 3D porous network interconnected by narrow windows with a diameter of 6 Å. The results obtained are similar to those of Sun *et al.* 2020.^14^ All tungsten doped samples show the same peaks characteristic of UiO-66; a comparison of the PXRD of UiO-66 and its derivatives shows that the crystal structure remains unchanged and that the incorporation of tungsten during the *in situ* reaction has not altered the structure of UiO-66, with the possibility of W–O bonding to Zr_6_O_4_(OH)_4_Fig. 1a.^38^ The typical reflections of the tungsten phases are not clearly observable in the PXRD analyses, but are more visible in the combined N_2_ and EDX sorption analyses due to the overlapping effect of the UiO-66 reflections. Nevertheless, a decrease in the relative intensity of the Bragg peaks of the UiO-66 diffraction of the simulated sample is observed, particularly at strong 2*θ* angles, with increasing tungsten concentration. Indeed, the change in electron density could be the cause of the decrease in intensity of the PXRD patterns. This confirms that the characteristic UiO-66 crystalline structure remains intact after the incorporation of W. Regarding the state and position of the tungsten (W) species, EDX elemental mapping indicates a homogeneous distribution of W within the UiO-66 particles. This suggests that the W species are well dispersed throughout the porous material, rather than forming large, separate crystalline phases. The absence of distinguishable W-related diffraction peaks in the PXRD patterns further suggests that the W species are highly dispersed or present in a non-crystalline form. PXRD of the by-products (Fig. 1b) of thermal degradation shows the formation of ZrO_2_ and hybrid oxides observable by the crystal phases of monoclinic WO_3_ at 2*θ* visible at 23–24° (ref. 36 and 37) and hexagonal WO_3_ at 2*θ* visible at 17.5° and 27.5°,^37,38^ more visible and intense for the ZrO_2_@WO_3_-60% sample.

**Fig. 1:**
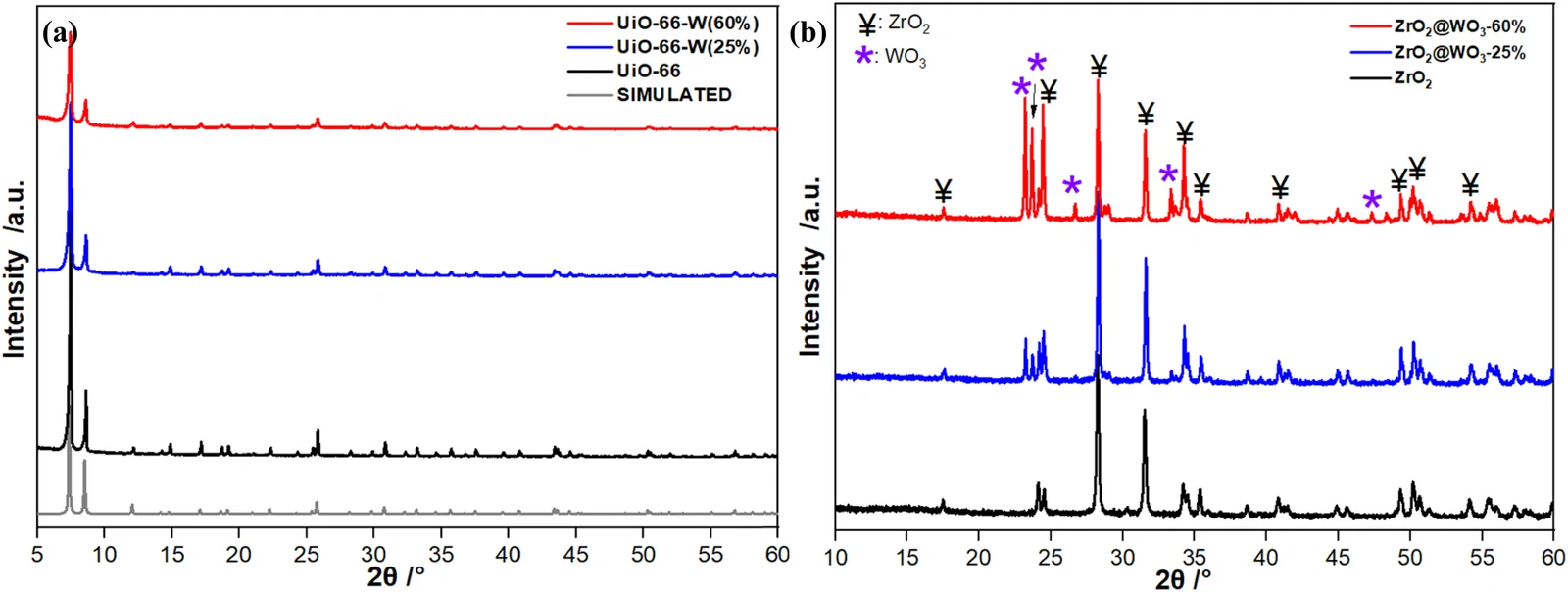
XRD patterns of UiO-66 and Zr/W-UiO-66(*x*) (a) and their by-products from the thermal decomposition ZrO_2_, ZrO_2_@WO_3_(*x*) with different W (*x* = 25 and 60%) concentration (b).

### 3.2 FT-IR analysis of UiO-66, Zr/W-UiO-66 and their thermal decomposition products

FTIR analyses of UiO-66, Zr/W-UiO-66 and it's thermal decomposition products ZrO_2_, ZrO_2_@WO_3_-25% and ZrO_2_@WO_3_-60% are shown in Fig. 2(a and b). From Fig. 2a, the strong bands at 2900 cm^−1^ present in the UiO-66 sample shows the presence of hydroxyl group attributed to the adsorbed O–H molecules. The vibrational peaks at 1650 cm^−1^ are those attributed to the C=O group of the BDC linker of the Zr/W-UiO-66 material. The peaks at 1585 cm^−1^ are due to the symmetric stretching bending modes of the O–C–O in the –COOH; the peak at 1510 cm^−1^ is due to the vibration of the C=C bonds in the BDC organic linker.^38^ The new calming bands at 1025 cm^−1^ can be attributed to tungsten hydrate associated with Zr_6_O_4_(OH)_4_ linked to 1,4-benzodicarboxylic acid. The broad and intense band at 1305 cm^−1^ is attributed to the *C*–O vibrational modes linked to the Zr_6_ node clusters.^42^ The small vibrations at 755 cm^−1^ and 640 cm^−1^ are those assimilated to the Zr–O–W and W–O–C vibrational modes in the structure of the materials^40,41^. Fig. 2b shows the formation of ZrO_2_ and ZrO_2_@WO_3_ hybrid oxides. The band at 2989 cm^−1^, present in all samples, can be assimilated to the stretching vibrations of the O–H groups of the water molecules superficially adsorbed in the monoclinic and hexagonal lattices of the ZrO_2_ and hybrid oxides.^42,43^ The presence of the band at around 755 cm^−1^ in the W-containing samples, alongside its absence or significantly lower intensity in pristine UiO-66, indicates that this vibration is related to the incorporation of W-containing species and can be attributed to Zr–O–W interactions.^44,45^ The band visible at 1700 cm^−1^ could be attributed to the carbonyl group present in the carboxylic acid of the linker. The adsorption peaks at 1376 cm^−1^ are attributed to the stretching vibrations of the O–Zr–O and O–W–O hybrid oxides. The intense adsorption band at 883 cm^−1^, the most visible for ZrO_2_@WO_3_-60%, is attributed to O-WO_3_ vibrations in the WO_3_ monoclinic phase,^46^ while those at 533 cm^−1^ can be assimilated to Zr-O-WO_3_ vibrational modes in hexagonal phases. The signal at 488 cm^−1^ can be attributed to the vibrational bonds of Zr–O.

**Fig. 2:**
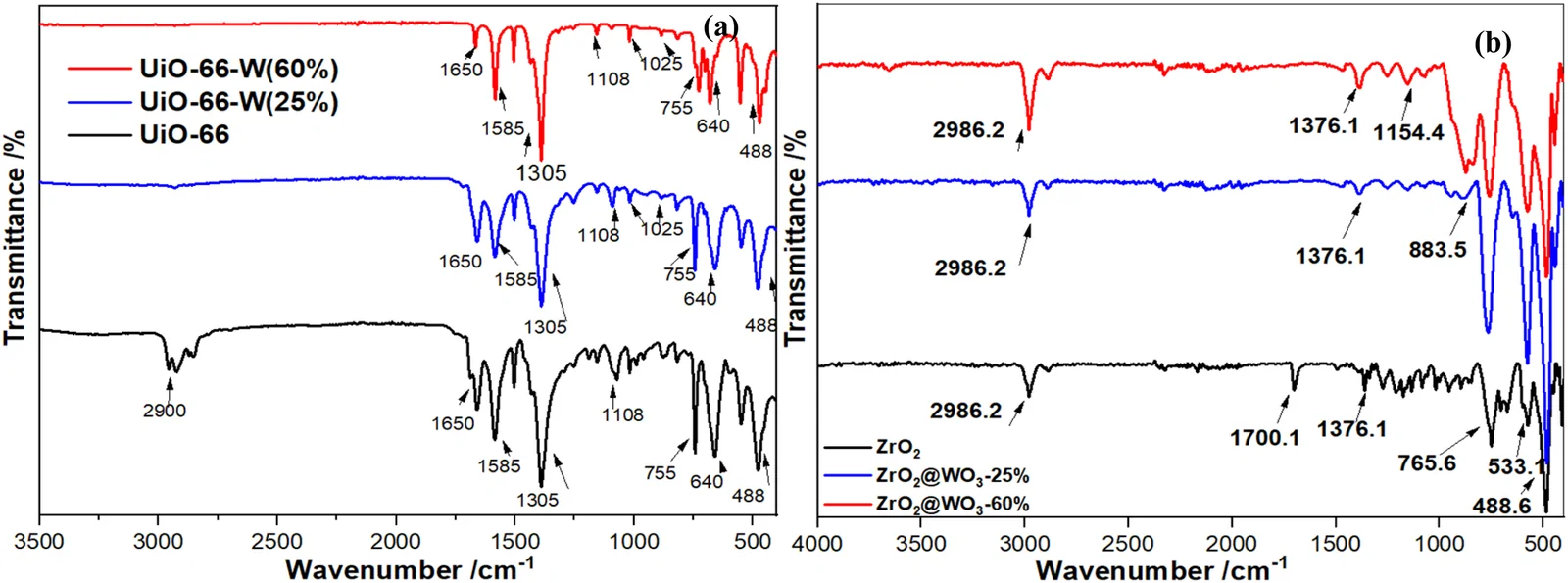
FTIR spectra of UiO-66 and Zr/W-UiO-66(*x*) (a) and their by-products from the thermal decomposition ZrO_2_, ZrO_2_@WO_3_(*x*) with different W (*x* = 25 and 60%) concentration (b).

### 3.3 SEM analysis of UiO-66, Zr/W-UiO-66 and their thermal decomposition products

SEM and EDX spectra of UiO-66, Zr/W-UiO-66 and its thermal decomposition products ZrO_2_, ZrO_2_@WO_3_-25% and ZrO_2_@WO_3_-60% are shown in Fig. 3(a and b). The SEM images (Fig. 3a) show the formation of UiO-66 by recombination of the bonds between the Zr_6_-node clusters and the –COOH group of the BDC linker with octahedral shaped particles.^38^ However, a progressive reduction in particle size is observed as the W concentration increases. Notably, the incorporation of 60 wt% W significantly restricts the growth of primary UiO-66 units into larger particles, resulting in smaller, less developed crystallites compared to pristine UiO-66. Increasing the concentration of W reduces the particle size of UiO-66, with 60 wt% of W added significantly inhibiting the growth of primary units into macromolecules; this could explain the reduced intensities of standard reflection peaks from the XRD pattern presented previously, a similar relationship was observed by Choi *et al.* 2021.^38^ Nevertheless, W species are observable and homogeneously distributed in the cavities of the UiO-66 framework (Fig. 3b), as observed by EDX mapping analyses. SEM analyses of the thermal decomposition clearly show the formation of zirconia nanoparticles (Fig. 3b), monoclinic and hexagonal WO_3_ phases in hybrid oxides (Fig. 3b),^40^ proof of the formation of Zr–O–W oxides^38,42^. Combined EDX and EDX elemental distribution mapping (Fig. S1) confirms the presence of tungsten and indicates its relatively homogeneous spatial distribution throughout the UiO-66 particles, supporting the successful incorporation of tungsten during solvothermal synthesis. The morphological evolution is also consistent with the textural results, where an increase in W content leads to a decrease in BET surface area and pore volume. This indicates a partial modification of the accessible porous structure. Following thermal treatment, SEM images reveal the formation of zirconia nanoparticles and W-containing oxide phases. The presence of monoclinic and hexagonal WO_3_ phases also supports the formation of Zr–O–W-containing hybrid oxides.^35,42^ This result shows that the solvothermal method promotes a homogeneous distribution of metal species throughout the framework of the material.

**Fig. 3:**
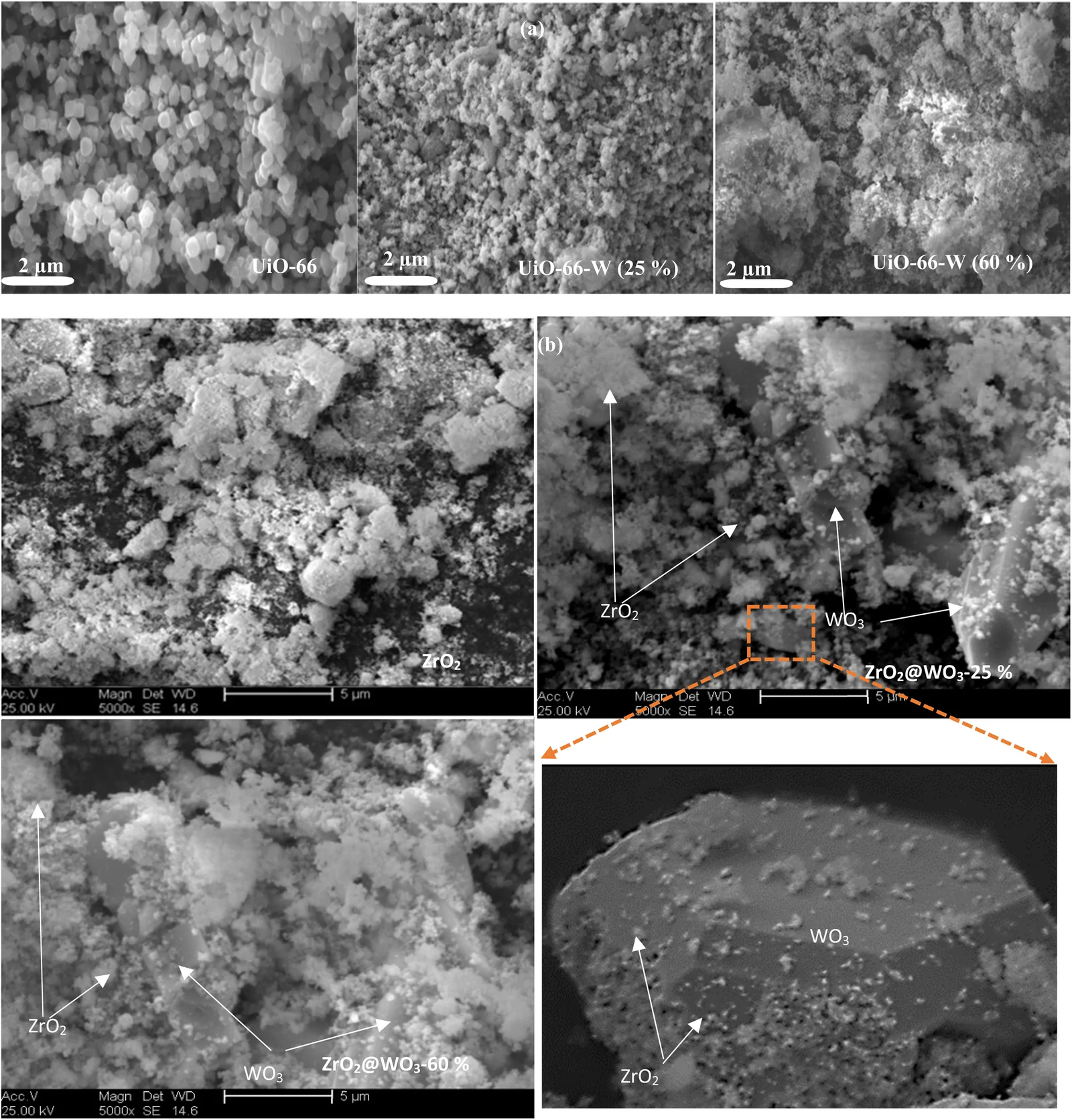
SEM images of UiO-66 and Zr/W-UiO-66(*x*) (a), SEM images of ZrO_2_, ZrO_2_@WO_3_(*x*) with different concentrations of W (*x* = 25 and 60%) (b), from the thermal decomposition at 10 K min^−1^.

### 3.4 N_2_-sorption analysis of UiO-66 and Zr/W-UiO-66

Fig. 4 shows the N_2_ adsorption and desorption isotherm curves for UiO-66, UiO-66-W (25%) and UiO-66-W (60%). The isotherms obtained are of type I without hysteresis. The N_2_ adsorption isotherms obtained are similar to those presented by Choi *et al.* in 2021.^38^ The pore size distribution (Fig. 4b) confirms the presence of micropore in the cavities of the sample lattice. All samples show a micropore size of around 100 nm with a narrow pore size distribution. The specific surface areas obtained are 1566, 1044 and 1002 m^2^ g^−1^ and the pore volumes are 0.4689, 0.4197 and 0.3922 cm^3^ g^−1^ for UiO-66, UiO-66-W (25%) and UiO-66-W (60%) respectively. The obtained BET surface areas are close to those obtained by Yang *et al.* in 2020.^14^ This reduction can be attributed to the introduction and distribution of W-containing species within the porous structure, which may partially occupy or block accessible pores, consequently reducing the accessible surface area and pore volume. The slight difference in BET surface area between UiO-66-W (25%) and UiO-66-W (60%) indicates that the majority of accessible porosity is retained, even at higher W loadings. These results indicate that the incorporation of W modifies the textural properties of UiO-66 while preserving its characteristic crystalline framework. The BET surface area of the samples decreases with increasing W concentration, although the pore size of the samples remains almost similar, suggesting that the presence of tungsten partially blocks some active entry sites for N_2_ molecules and the collapse of UiO-66 micropores after W incorporation.^38^ Nevertheless, the BET surface area remains relatively uniform and comparable to those previously obtained.^47^

**Fig. 4:**
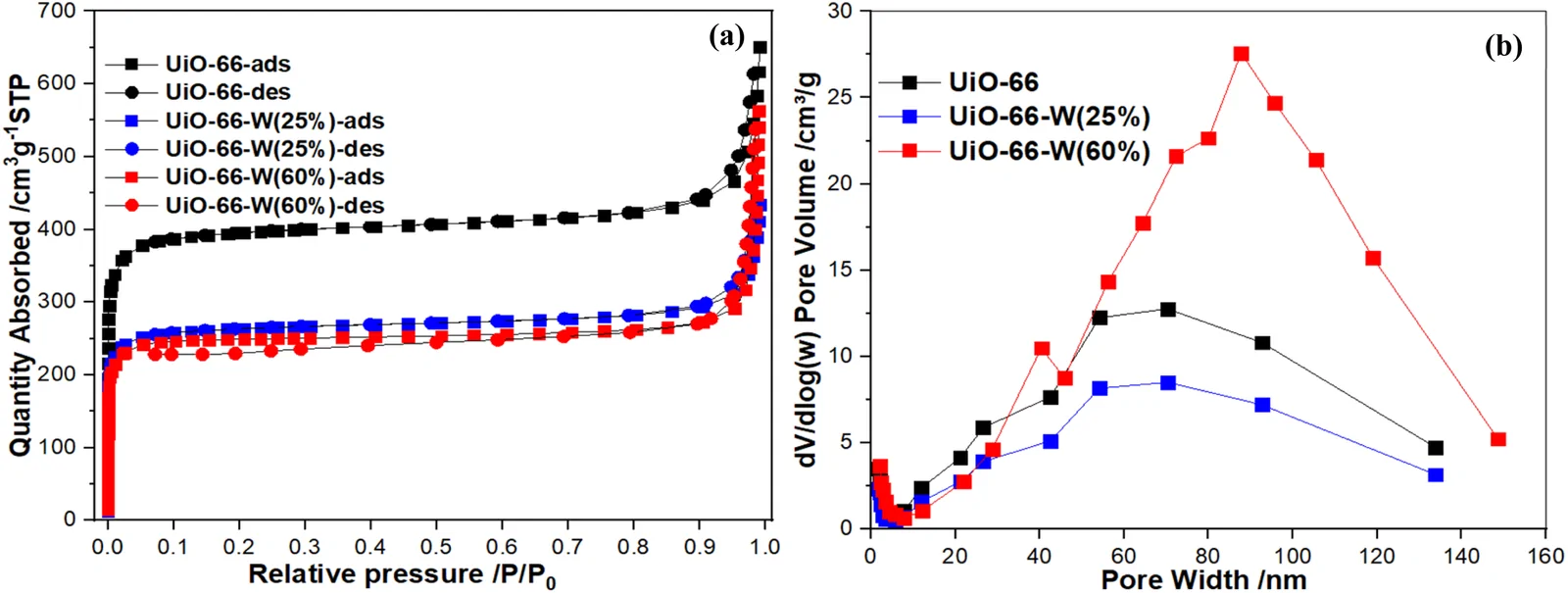
N_2_ adsorption/desorption isotherm (a) and pore size distribution (b) of UiO-66 and Zr/W-UiO-66(*x*) with different W (*x* = 25 and 60%) concentration.

### 3.5 Thermal decomposition analysis and kinetic of UiO-66 and Zr/W-UiO-66

#### 3.5.1 TGA, DTA and DTG analysis

Fig. 5(a–d) show the TGA/DTA/DTG curves of UiO-66, UiO-66-W (25%) and UiO-66-W (60%) at 10 K min^−1^ and the effect of heating rate. Fig. 5b shows that the thermal decomposition of UiO-66 occurs in two main stages, for temperatures between 25 and 400 °C and 400 and 730 °C for steps 1 and 2, respectively. The TGA curve of the UiO-66 sample obtained is similar to that of Shearer *et al.* 2016.^5^ Three mass losses can be observed on the TGA curves, the first loss in the temperature range from 25 to 250 °C is due to the loss of water molecules, methanol and intra-framework DMF, the second mass loss between 250 and 450 °C is due to the loss of carboxylate ligands and the dehydroxylation of Zr_6_O_4_(OH)_4_ secondary building blocks.^5^ The third mass loss above 550 °C is due to the decomposition of the UiO-66 structure and the formation of Zr_6_O_6_(BDC)_6_, accompanied by an intense exothermic peak; above 700 °C the Zr_6_O_6_(BDC)_6_ transforms into ZrO_2_, followed by the formation of the platform observed on the TGA curve of the UiO-66 material. Similar reports have been presented by Shearer *et al.* 2016.^5,14^

**Fig. 5:**
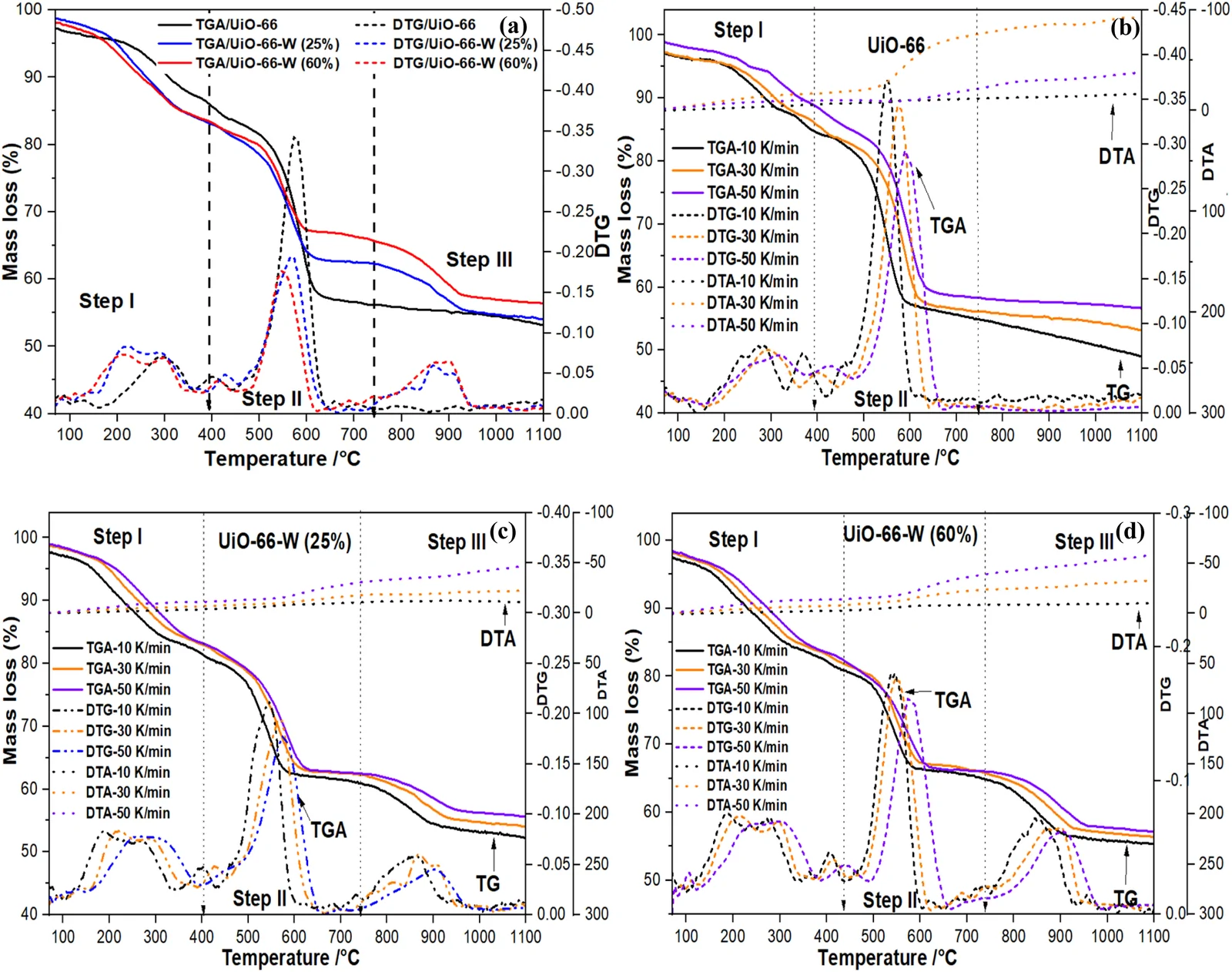
TGA/DTG curves of UiO-66 and Zr/W-UiO-66 at 10 K min^−1^ (a), TGA/DTA/DTG behavior of UiO-66 (b), UiO-66-W (25%) (c) and UiO-66-W (60%) (d), at 10, 30 and 50 K min^−1^.

For Zr/W-UiO-66 samples Fig. 5(c and d) the TGA curves are almost similar and occur in three steps, for temperatures between 25–400 °C, 400–730 °C and 730–1100 °C for steps 1–3 respectively. The difference in mass loss observed for UiO-66-W (25%) and UiO-66-W (60%) is due to the difference in W concentration in the two materials. However, in addition to the three mass losses observed in the case of UiO-66 material, the DTG curves of UiO-66 material are more significant than those of Zr/W-UiO-66, indicating the presence of W. The DTG profiles for Zr/W-UiO-66 samples show more significant mass losses in the temperature range of 25 to 250 °C, indicating the presence of water and ethanoate molecules bound to ammonium metatungstate hydrate.^38^ The step 2 for the temperature range of 400 to 750 °C allows a quantitative comparison of the mass losses of UiO-66 and Zr/W-UiO-66 samples, this step can be observed by the more pronounced vertical line (Fig. 5a), at this stage we observe that the magnitude of the decomposition mass loss of the UiO-66 samples is more significant compared to that of the Zr/W-UiO-66 samples, indicating that the Zr/W-UiO-66 samples are deficient in BDC linker compared to the UiO-66 samples. More importantly, a different behavior is observed above 750 °C, showing the presence of W in the framework of the Zr/W-UiO-66 samples. The presence of W can be confirmed by the fourth mass loss in the range 750 to 1100 °C (Fig. 5a) in step 3, due to the decomposition of W_12_O_40_ to WO_3_ and the formation of Zr–O–W hybrid oxides, as observed in previous XRD analyses. The mass loss events are also associated with the decomposition of the framework of each sample involving complete breakdown of the BDC linkers connected to the Zr_6_O_4_(OH)_4_ clusters.^11,14^ This high-temperature event is attributed to the transformation/decomposition of tungsten-containing species, resulting in the formation of WO_3_ and Zr–O–W oxide phases. This is in line with the XRD results obtained after thermal treatment. The persistence of this high-temperature transformation suggests that tungsten-containing species significantly alter the thermal decomposition pathway of UiO-66, contributing to the formation of thermally stable Zr/W oxide phases. Furthermore, increasing the W content from 25 to 60 wt% alters the extent of the observed mass losses, confirming the impact of W loading on thermal decomposition behavior. Thus, the TGA/DTG results demonstrate that the incorporation of W does not simply increase or decrease the thermal stability of UiO-66 in a single step, but rather modifies the decomposition pathway, particularly at high temperatures, by introducing additional W-containing species and favoring the formation of Zr–O–W hybrid oxide products.

Fig. 5(b–d) shows the effect of heating rate at 10, 30 and 50 K min^−1^ during the thermal decomposition of UiO-66, UiO-66-W (25%) and UiO-66-W (60%), respectively. All TGA curves are similar and show the same sample-dependent concavities with DTG derivative peak shifts.^48^ From Fig. 5b, a DTG peak shift can be observed at temperatures of 255.8, 268.2 and 278.7 °C for step 1 and 554.2, 569.4 and 588.4 °C for step 2, measured for the UiO-66 sample treated at 10, 30 and 50 K min^−1^, respectively.^49^ Similar observations can be observed for the Zr/W-UiO-66 samples. The UiO-66-W (25%) samples show a variations in DTG peaks at temperatures of 196.5, 212.2 and 238.7 °C for step 1, 552.5, 564.5 and 580.2 °C for step 2 and 860.3, 872.8 and 890.3 °C for stage 3 at heating rates of 10, 30 and 50 K min^−1^, these variations can be explained by the reduction in the heat of absorption by diffusion due to the high heating rates of 30 and 50 K min^−1^;^17,21^ the reduction in the energy supplied by the system causes a thermal variation between the sampling frames of the outermost molecules and those located towards the crystal cores.^46,47^ Nevertheless, the whole process leads to the formation of ZrO_2_ and hybrid oxides *via* the recombination of dehydroxylated Zr_6_O_6_ and W_12_O_40_.^39,40^ The influence of the heating rate shows an exponential and linear dependence on the peaks of the DTG curves and the heating temperature.^18,21^ The formation of ZrO_2_ and hybrid oxides can be deduced from the similarity of the TGA curves above 800 °C. The data from the TGA and DTG curves were used for thermokinetic analysis.

#### 3.5.2 Thermokinetic study of the decomposition of the UiO-66 and Zr/W-UiO-66 samples

##### 3.5.2.1 Determination of *E*_a_ by FWO, KAS, FWO-Ir and KAS-Ir methods

The activation energy (*E*_a_) was evaluated on the basis of heating rates of 10, 30 and 50 K min^−1^. The integral methods of FWO and KAS were used as a function of the conversion rate. The *E*_a_ for steps 1 and 2 for UiO-66 and those for steps 1, 2 and 3 of the thermal decomposition of UiO-66-W (25%) and UiO-66-W (60%) were determined from the slope of the plot of ln(*β*/*T*_*α*_^2^) against 1000/*T*_α_ for the KAS method Fig. S2(a, b), S3(a–c) and S4(a–c); and ln(*β*) *versus* 1000/*T*_α_ for the FWO method Fig. S2(c, d), S3(c–e) and S4(c–e) in SI using eqn (9) and (10), respectively.^51^ Table S2 shows the first values of the activation energy obtained as a function of the degree of conversion (*α*) from 0.1 to 0.9 for the FWO and KAS models.^52^ For the UiO-66 sample, the average *E*_a_ were 124 and 112 kJ mol^−1^; 276 and 269 kJ mol^−1^ for the FWO and KAS methods for steps 1 and 2, respectively. For the UiO-66-W (25%) sample, the average *E*_a_ were 115 and 102 kJ mol^−1^; 256 and 249 kJ mol^−1^; and 334 and 327 kJ mol^−1^ for the FWO and KAS methods for steps 1, 2 and 3, respectively. For the UiO-66-W (60%) sample, the mean *E*_a_ were 85 and 71 kJ mol^−1^; 239 and 231 kJ mol^−1^; and 358 and 351 kJ mol^−1^ for the FWO and KAS methods for steps 1, 2 and 3, respectively, as shown in Tables S2–S4. From Tables S2–S4, it can be observed that the *E*_a_ obtained with the FWO method were higher than those obtained with the KAS method; for example, the average *E*_a_ obtained with the FWO method showed differences (*Δ* ≈ 6–13 kJ mol^−1^) comparable to those obtained with the KAS method, which shows the lack of accuracy of the model used and the unreliability of the integral approximation of the conversion temperatures, hence the need for an iterative procedure.^50,51^ Furthermore, the average *E*_a_ for the UiO-66-W (60%) sample for step 3 were found to be higher than those for the UiO-66-W (25%) sample. In parallel, the average *E*_a_ obtained from the UiO-66 sample for steps 1 and 2 were higher, comparable to those of the Zr/W-UiO-66 samples, indicating the presence of metal W in the framework of the UiO-66 sample.^54^ The difference between the average *E*_a_ obtained could be explained by the low mass loss, as observed in the TGA curves, and the variation in the conversion rate (*α*); the low conversion rates presented small values of *E*_a_, due to the low heating temperatures that supply low energies to the system. Furthermore, the difference in the average *E*_a_ obtained for the Zr/W-UiO-66 samples, could be explained by the difference in the metal W concentration in the samples; the decomposition of the W framework into WO_3_, was observed above 800 °C (step 3), the high metal W concentration could reduce the total mass loss, as observed in the UiO-66-W (60%) sample.

*E*_a_ were calculated using the iterative procedure of the FWO-Ir and KAS-Ir methods from the slope of the plot of ln(*β*/*h*(*x*)*T*_*α*_2) as a function of 1000/Tα for the KAS-Ir method Fig. 6(a and b), for steps 1 and 2 for UiO-66 sample; Fig. 6(a-1, b-1, c-1, a-2, b-2 and c-2) for steps 1, 2 and 3 for Zr/W-UiO-66; and ln(*β*/*H*(*x*)) *versus* 1000/Tα for the FWO-Ir method Fig. 6(c and d), for steps 1 and 2 for UiO-66 sample; Fig. 6(d-1, e-1, f-2, d-2, e-2 and f-2) for steps 1, 2 and 3 for Zr/W-UiO-66 sample using eqn (11) and (12), respectively.

**Fig. 6:**
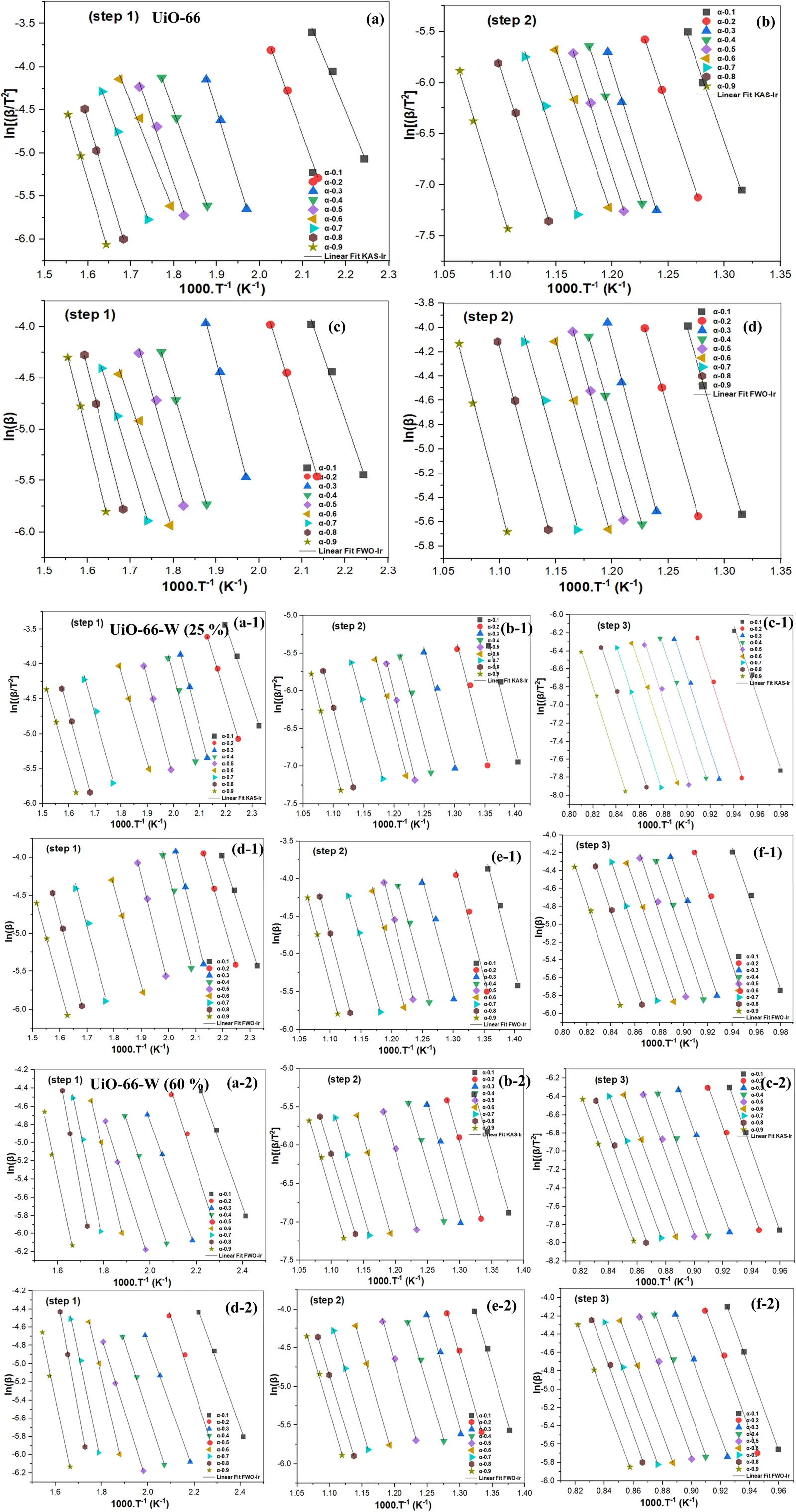
KAS-Ir (a and b) and FWO-Ir methods (c and d) for steps 1 and 2, respectively for the UiO-66 sample. KAS-Ir (a-1, b-1 and c-1) and FWO-Ir methods (d-1, e-1 and f-1) for steps 1, 2 and 3, respectively for UiO-66-W (25%) sample. KAS-Ir (a-2, b-2 and c-2) and FWO-Ir method (d-2, e-2 and f-2) for steps 1 and 2, respectively for UiO-66-W (60%) sample.

The obtained activation energies and regression coefficients (*R*^2^) are presented in Tables S5–S7 for samples UiO-66, UiO-66-W (25%) and UiO-66-W (60%), respectively. According to these tables, the mean *E*_a_ for sample UiO-66 were 120.87 and 120.83 kJ mol^−1^; 279.74 and 279.69 kJ mol^−1^ for steps 1 and 2 using the FWO-Ir and KAS-Ir methods, respectively. In addition, for sample UiO-66-W (25%), the *E*_a_ were 111.59 and 111.54 kJ mol^−1^; 258.97 and 258.92 kJ mol^−1^; 339.33 and 339.27 kJ mol^−1^ for steps 1–3 using the FWO-Ir and KAS-Ir methods, respectively. The *E*_a_ for sample UiO-66-W (60%) were 80.54 and 80.51 kJ mol^−1^; 241.58 and 241.53 kJ mol^−1^; 363.76 and 363.70 kJ mol^−1^ for steps 1, 2 and 3 using the FWO-Ir and KAS-Ir methods, respectively. The average *E*_a_ obtained by the FWO-Ir and KAS-Ir methods are all close to each other (*Δ* ≈ 0.1 kJ mol^−1^). The values obtained by the FWO-Ir and KAS-Ir methods are comparable to the *E*_a_ obtained by the FWO and KAS methods, which show variations greater than 10 kJ mol^−1^. These observations show that the fourth approximation of Serum and Yang^29,53^ is suitable for evaluating the most accurate values of *E*_a_. This is confirmed by regression coefficients greater than 0.99, *R*^2^ close to unity, confirming the reliability of the FWO-Ir and KAS-Ir iterative procedures adopted. It can be seen that, the KAS and FWO methods cannot be used without the iterative correction procedure.^56^ On the other hand, the iterative method is more reliable and provides an improvement in *E*_a_. From Fig. 7(a–c), it can be seen that the *E*_a_ values depend on the degree of conversion *α*. This observation shows that the thermal decomposition of Zr/W-UiO-66 samples is not uniform and the degradation process is governed by several reaction mechanisms.^55,56^ Chen *et al.* 2012 (ref. 51) showed that mechanisms with multiple reaction steps are dominated by a variation in activation energy.^57,58^ In addition, the increase in *E*_a_ beyond the conversion rate of 0.5 indicates the existence of a complex process during the thermal decomposition of Zr/W-UiO-66 involving multiple chemical mechanisms.^59^

**Fig. 7:**
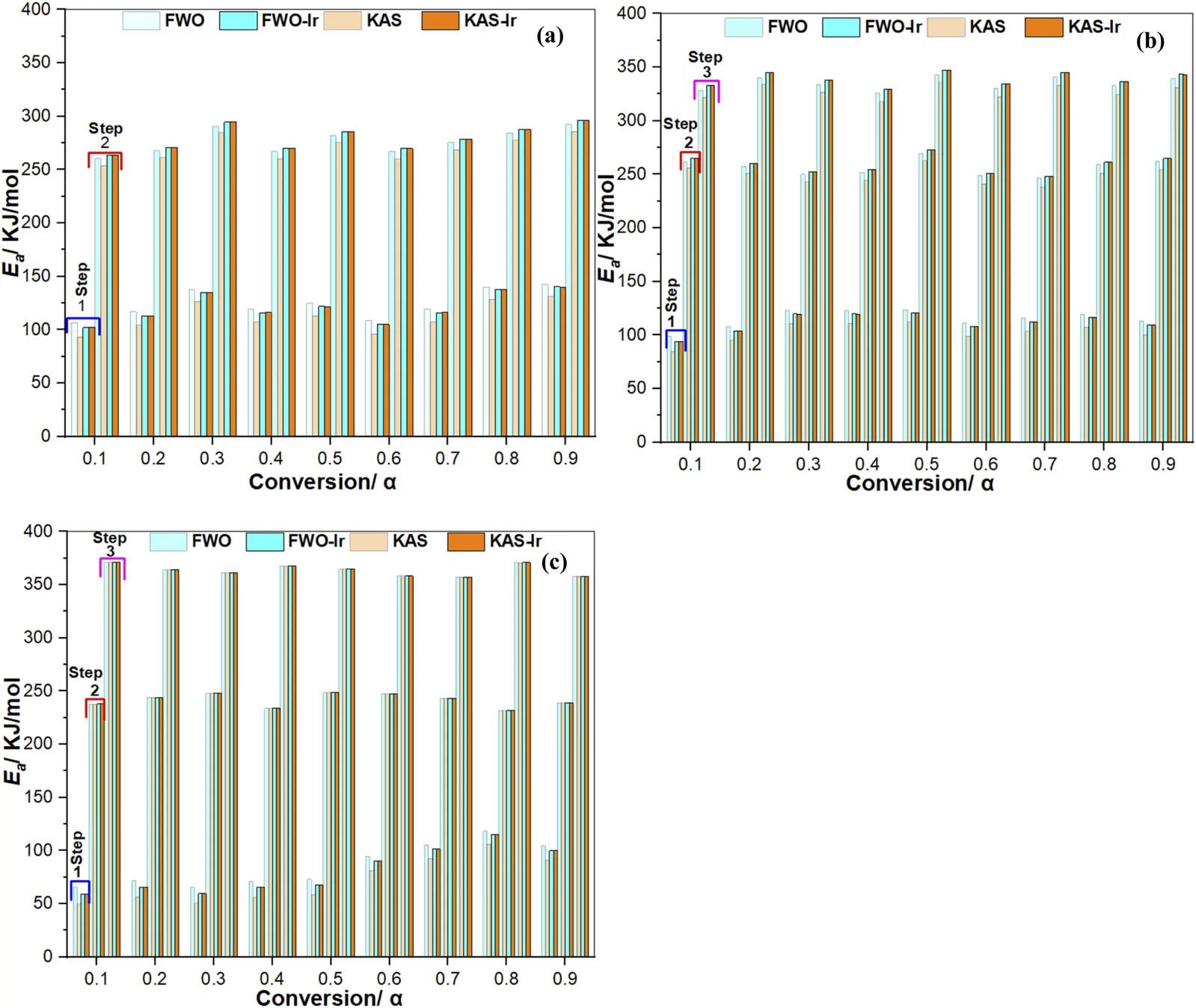
*E*_a_ *versus* degree of conversion *α* for the thermal degradation of UiO-66 (a), UiO-66-W (25%) (b) and UiO-66-W (60%) (c), for steps 1–3, calculated using the FWO and KAS methods, as well as the FWO-Ir and KAS-Ir methods.

#### 3.5.3 Determination of the most probable mechanism function *g*(*α*)

The mechanism function *g*(*α*) of the Zr/W-UiO-66 samples was determined by the multiple heating scan rate method, using the plot of *lng*(*α*) *vs.* ln(*β*) Fig. S5 for UiO-66 sample and Fig. 8(a–f) for steps 1–3 for Zr/W-UiO-66. The conversion rates (*α*) corresponding to each heating rate and the same temperature were used. The *α* values used, corresponding to *β* = 10, 30 and 50 K min^−1^, are listed in Table S8; 36 types of functions (Table S1) were tested for this purpose.^18,21^ Table S9 shows the most probable *g*(*α*) functions. Functions G5, F2, F3, P3/2, D7, D8, G6, G4, G5, D5, F4, D3 and D4 were found to have the highest regression coefficients (*R*^2^). Meanwhile, G5 and D8 functions for the UiO-66 samples showed the slope closest to −1 for steps 1 and 2, respectively. Similarly, G5, G5 and D5 for the UiO-66-W (25%) samples; G5, P3/2 and D3 showed the slope closest to −1 and *R*^2^ closest to 1 for steps 1, 2 and 3 respectively compared to the other models. This shows that the thermal decomposition of UiO-66, UiO-66-W (25%) and UiO-66-W (60%) is complex reaction, proceeds in several steps and is governed by an assumed random nucleation mechanism and its subsequent growth (G5), followed by a three-dimensional diffusion mechanism (D8 or D5), a nucleation mechanism (P3/2) and three-dimensional diffusion (D3).^15,16^

**Fig. 8:**
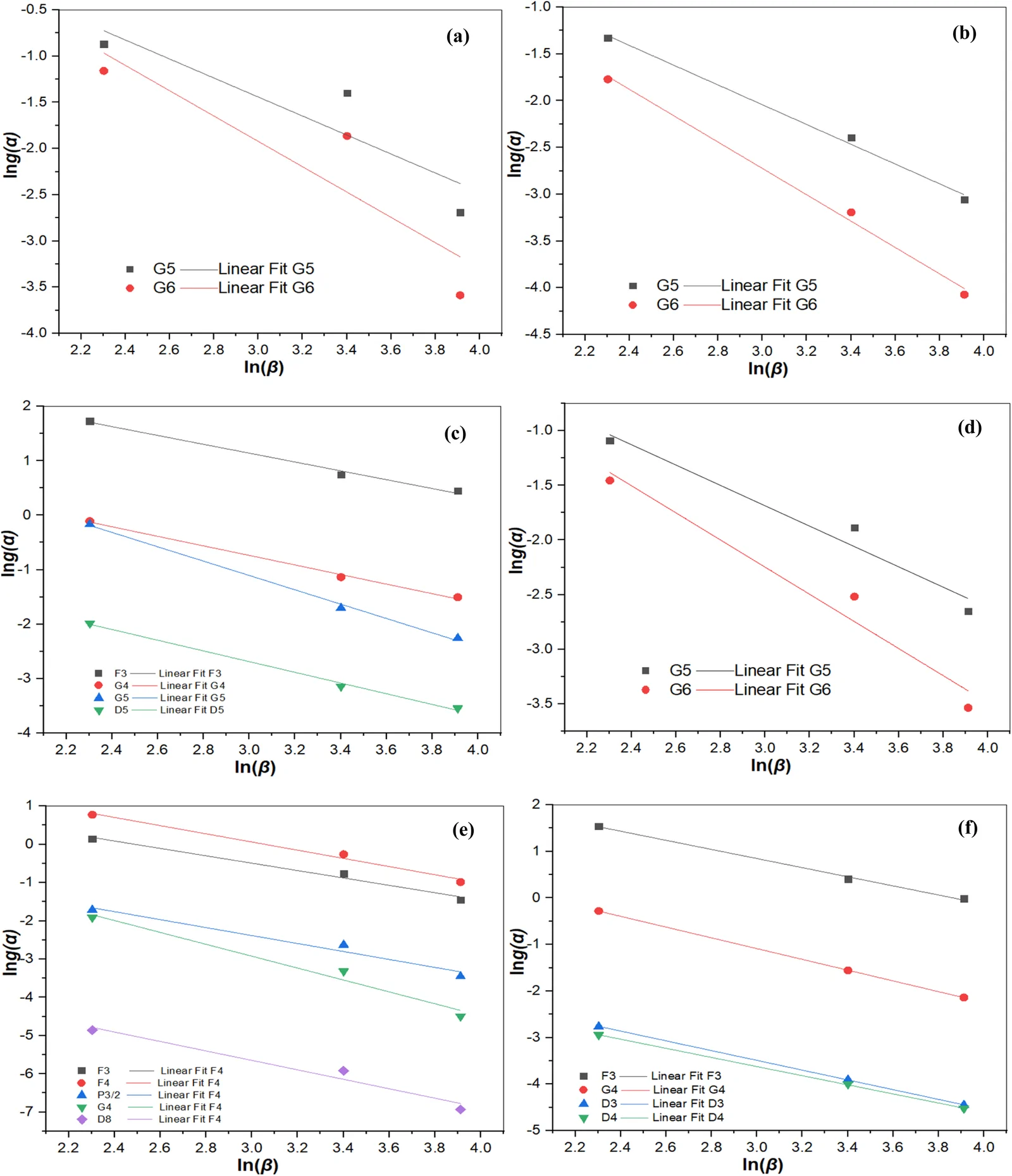
Plot of ln *g*(*α*) *versus* ln(*β*) for UiO-66-W (25%) (a–c) and for UiO-66-W (60%) (d–f) for steps 1–3 of the thermal degradation, respectively.

#### 3.5.4 Determination of the pre-exponential factor (*A*)

The pre-exponential factor was calculated from the intercept of the plots of eqn (11) for the FWO-Ir models and eqn (12) for the KAS-Ir models, using the *E*_a_ corresponding to each degree of conversion (*α*) obtained by the iterative procedure and the most probable *g*(*α*).^60^ The mean value of *A* for UiO-66: 2.29 × 10^12^ and 7.13 × 10^11^ min^−1^; 4.04 × 10^15^ and 3.88 × 10^15^ min^−1^ for FWO-Ir and KAS-Ir corresponding to steps 1 and 2, respectively. For UiO-66-W (25%): 6.80 × 10^11^ and 1.57 × 10^11^ min^−1^; 9.69 × 10^15^ and 8.10 × 10^15^ min^−1^; 1.88 × 10^14^ and 1.44 × 10^14^ min^−1^ for FWO-Ir and KAS-Ir according to steps 1–3 respectively. For UiO-66-W (60%): 1.39E10 and 2.96 × 10^9^ min^−1^; 1.31 × 10^15^ and 9.78 × 10^14^ min^−1^; 9.98 × 10^14^ and 9.03 × 10^14^ min^−1^, for FWO-Ir and KAS-Ir according to steps 1–3, respectively. As shown in (Tables S5–S7), the *A* value increases reciprocally with temperature, *e.g.* the UiO-66 sample shows a pre-exponential factor of 2.29 × 10^12^ min^−1^ and 4.04 × 10^15^ min^−1^ for average temperatures of 278 °C and 550 °C corresponding to steps 1 and 2, respectively; similar observations were found for Zr/W-UiO-66 samples. In parallel, the pre-exponential factors obtained from the FWO and KAS models decrease with increasing conversion rate (*α*). Small values of A for *α* > 0.5 indicate low reactivity.^61^ On the other hand, large values of *A* for *α* < 0.5 indicate the presence of complex and more reactive reactions, suggesting that thermal decomposition of Zr/W-UiO-66 samples at high temperatures is spontaneous due to complex reactions.^32,59^

### 3.6 Determination of the thermodynamic variables (Δ*S*^≠^, Δ*H*^≠^, Δ*G*^≠^)

The thermodynamic variables obtained from the FWO-Ir and KAS-Ir models are summarized in Tables S10–S12. Δ*S*≠ for UiO-66: −42.84 and −54.88 J mol K^−1^; 28.96 and 28.96 J mol K^−1^ for FWO-Ir and KAS-Ir according to steps 1 and 2, respectively. For UiO-66-W (25%): −46.72 and −60.36 J mol K^−1^; 39.68 and 37.49 J mol K^−1^; 6.35 and 4.25 J mol K^−1^ for FWO-Ir and KAS-Ir according to steps 1, 2 and 3 respectively. For UiO-66-W (60%): −106.61 and −126.14 J mol K^−1^; 17.78 and 14.35 J mol K^−1^; 22.02 and 21.11 J mol K^−1^, for FWO-Ir and KAS-Ir according to steps 1, 2 and 3, respectively, were obtained. The obtained Δ*S*^≠^ decrease with increasing decomposition temperature due to the presence of complex and disordered reactions.^19^ For step 1, the Δ*S*^≠^ < 0 for FWO-Ir and KAS-Ir shows the formation of an activated complex during the dehydration step and the departure of the solvent in Zr/W-UiO-66 samples with a more ordered transition state. While the Δ*S*^≠^ > 0 for steps 2 and 3 are related to the dehydroxylation of the secondary building units and the decomposition of the structure in the Zr/W-UiO-66 samples due to the formation of an ordered structure of the activated complex during the recombination step of Zr_6_O_6_ and W_12_O_40_.^17,21^ Furthermore, Δ*H*^≠^ and Δ*G*^≠^ were found to increase reciprocally with temperature and conversion rate (*α*) for all Zr/W-UiO-66 samples. The variation of Δ*H*^≠^ could be explained by the energy change supplied in to the system during the reaction due to the variation of the heating rate and the temperature. Furthermore, Δ*H*^≠^ > 0 is materialized by the presence of endothermic peaks in the DTA curves. While Δ*G*^≠^ > 0 characterizes the stability of the active ZrO_2_ complex and hybrid oxides during the recombination process of dehydroxylated Zr_6_O_6_. Furthermore, Δ*G*^≠^ > 0 implies that the dehydroxylation of the –OH groups of Zr_6_O_4_(OH)_4_ clusters and the recombination of WO_3_ are not spontaneous at low temperatures.^62^ It is also observed that steps 2 and 3 are more difficult to achieve than the first step, which requires little energy. The higher Δ*H*^≠^ and Δ*G*^≠^ for UiO-66-W (25%) and UiO-66-W (60%) for conversion rates (*α* > 0.6) can be explained by the maximum decomposition, as well as by the presence of the metal tungsten, which would provide more energy to the system due to the diffusion process between the binding energies of the –OH groups and the oxygen atoms of W_12_O_40_. Fig. 9 shows the average values of Δ*S*^≠^, Δ*H*^≠^ and Δ*G*^≠^ from the FWO-Ir and KAS-Ir modes. The plots show an approximation between the mean values of Δ*S*^≠^, Δ*H*^≠^ and Δ*G*^≠^ for both models, confirming the accuracy of the *E*_a_ obtained using the iterative FWO-Ir and KAS-Ir procedure and the most likely mechanism functions.

**Fig. 9:**
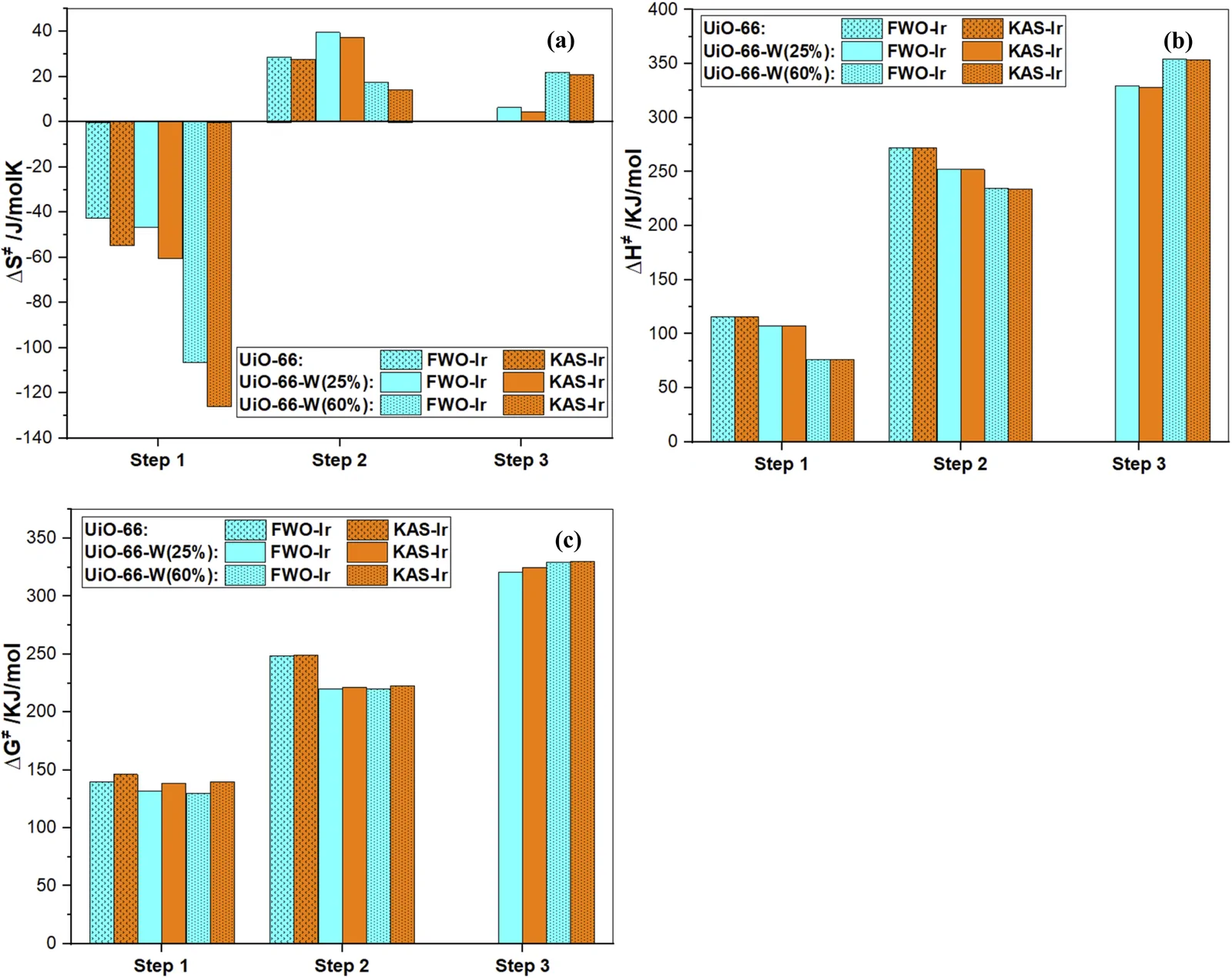
Bar charts showing the average thermodynamic parameters Δ*S*^≠^(a), Δ*H*^≠^(b) and Δ*G*^≠^(c) of the thermal degradation of Zr/W-UiO-66 using *E*_a_ derived from either KAS-Ir or FWO-Ir methods.

## 4 Conclusion

The incorporation of tungsten into the UiO-66 structure was carried out *in situ*, and several characterization techniques by PXRD, SEM, EDX and FT-IR were carried out on the samples. These techniques revealed the presence of tungsten with a homogeneous distribution in the cavities of UiO-66, as shown by the EDX elemental distribution mapping analyses. The thermal decomposition of the Zr/W-UiO-66 samples was studied using TGA analyses at heating rates of 10, 30 and 50 K min^−1^ over the temperature range of 25 to 1100 °C in an air atmosphere and the following information was observed:

• From TGA and DTG analyses, the thermal decomposition of Zr/W-UiO-66 occurs in three steps at temperatures between 25–400 °C, 400–730 °C and 730–1100 °C for steps 1–3, comparable to UiO-66 which occurs in two steps. The difference in mass loss observed for Zr/W-UiO-66 was attributed to the difference in W concentration.

• The effect of heating rate was observed by the shift of DTG peaks in the temperatures for each step, indicating the decrease in diffusion absorption heat due to high heating rates.

• The kinetic parameters of thermal decomposition, such as activation energy (*E*_a_) and pre-exponential factor, were evaluated using the iterative procedure of FWO-Ir and KAS-Ir. The average *E*_a_ obtained were close to each other (*Δ* ≈ 0.1 kJ mol^−1^). Both models proved to be adequate for the determination of *E*_a_, showing that the degradation process is governed by several reaction mechanisms.

• Thermokinetic analyses showed that the decomposition of the Zr/W-UiO-66 structure is governed by the following mechanisms: assumed random nucleation and subsequent growth (*G*_5_), three-dimensional diffusion (*D*_8_) and three-dimensional spherically symmetric diffusion (*D*_3_).

• The entropy change ((Δ*S*^≠^), enthalpy change (Δ*H*^≠^) and Gibbs free energy (Δ*G*^≠^) obtained showed lower derivations *Δ* ≈ 6% for steps 1–3, indicating the presence of complex and disordered reactions during dehydroxylation.

• The formation of ZrO_2_ and hybrid oxides was appreciated by the similarity of the TGA curves above 800 °C and was found to be reliable as a heterogeneous catalyst for photocatalytic applications.

## Author contributions

Cyrille Ghislain Fotsop: writing – original draft, visualization, validation, resources, methodology, investigation, formal analysis, data curation, conceptualization. Alexandra Lieb: validation, supervision, investigation, formal analysis, data curation, writing – review & editing. Franziska Scheffler: validation, supervision, resources, funding acquisition, project administration.

## Conflicts of interest

The authors declare no conflicts of interest.

## Supplementary Material

RA-016-D6RA04868D-s001

## Data Availability

Data are available on request. Supplementary information (SI) is available. See DOI: https://doi.org/10.1039/d6ra04868d.
