# Supplementary material for: Non-isothermal TGA, mechanism and thermodynamic study of the thermal degradation of mixed metal Zr/W-UiO-66-type MOF materials
Source: RSC Adv. 2026 Oct 3;16(46):59861–76. doi: 10.1039/d6ra04868d (PMC13633110; doi:10.1039/d6ra04868d)
Supplement: Supplementary file 1 [file RA-016-D6RA04868D-s001.pdf]

Supplementary Information (SI) for RSC Advances.
This journal is © The Royal Society of Chemistry 2026
Supplementary Information (SI) for RSC Advances.
This journal is © The Royal Society of Chemistry

Supporting Information

Non-isothermal TGA, mechanism and thermodynamic study of the thermal degradation of mixed metal Zr/W-UiO-66-type MOF materials

Tab. S1: Algebraic expressions of functions $g(\alpha)$, $f(\alpha)$ and its corresponding mechanism [1, 2]

No	Symbol	$g(\alpha)$	$f(\alpha)$	Rate-determining mechanism
**1. Chemical process or mechanism - non-invocative equations**				
1	$F_{1/3}$	$1-(1-\alpha)^{2/3}$	$3/2\,(1-\alpha)^{1/3}$	Chemical reaction
2	$F_{3/4}$	$1-(1-\alpha)^{1/4}$	$4\,(1-\alpha)^{3/4}$	Chemical reaction
3	$F_{3/2}$	$(1-\alpha)^{-1/2}-1$	$2\,(1-\alpha)^{3/2}$	Chemical reaction
4	F_2	$(1-\alpha)^{-1}-1$	$(1-\alpha)^2$	Chemical reaction
5	F_3	$(1-\alpha)^{-2}-1$	$1/2\,(1-\alpha)^3$	Chemical reaction
6	F_4	$(1-\alpha)^{-3}-1$	$1/3\,(1-\alpha)^4$	Chemical reaction
7	G_1	$1-(1-\alpha)^2$	$1/2\,(1-\alpha)$	Chemical reaction
8	G_2	$1-(1-\alpha)^3$	$1/3\,(1-\alpha)^2$	Chemical reaction
9	G_3	$1-(1-\alpha)^4$	$1/4\,(1-\alpha)^3$	Chemical reaction
**2. Acceleration rate equations**				
10	$P_{3/2}$	$\alpha^{3/2}$	$(2/3)\,\alpha^{-1/2}$	Nucleation
11	$P_{1/2}$	$\alpha^{1/2}$	$2\,\alpha^{1/2}$	Nucleation
12	$P_{1/3}$	$\alpha^{1/3}$	$3\,\alpha^{2/3}$	Nucleation
13	$P_{1/4}$	$\alpha^{1/4}$	$4\,\alpha^{3/4}$	Nucleation
14	E_1	α	$\ln\alpha$	Nucleation
**3. Sigmoidal rate equations or random nucleation and subsequent growth**				
15	A_1, F_1	$-\ln(1-\alpha)$	$1-\alpha$	Assumed random nucleation and its subsequent growth
16	$A_{3/2}$	$[-\ln(1-\alpha)]^{2/3}$	$3/2\,(1-\alpha)\,[-\ln(1-\alpha)]^{1/3}$	Assumed random nucleation and its subsequent growth
17	A_2	$[-\ln(1-\alpha)]^{1/2}$	$2\,(1-\alpha)\,[-\ln(1-\alpha)]^{1/2}$	Assumed random nucleation and its subsequent growth
18	A_3	$[-\ln(1-\alpha)]^{1/3}$	$3\,(1-\alpha)\,[-\ln(1-\alpha)]^{2/3}$	Assumed random nucleation and its subsequent growth
19	A_4	$[-\ln(1-\alpha)]^{1/4}$	$4\,(1-\alpha)\,[-\ln(1-\alpha)]^{3/4}$	Assumed random nucleation and its subsequent growth
20	G_4	$[-\ln(1-\alpha)]^2$	$1/2\,(1-\alpha)\,[-\ln(1-\alpha)]^{-1}$	Assumed random nucleation and its subsequent growth
21	G_5	$[-\ln(1-\alpha)]^3$	$1/3\,(1-\alpha)\,[-\ln(1-\alpha)]^{-2}$	Assumed random nucleation and its subsequent growth
22	G_6	$[-\ln(1-\alpha)]^4$	$1/4\,(1-\alpha)\,[-\ln(1-\alpha)]^{-4}$	Assumed random nucleation and its subsequent growth
23	Au	$\ln\alpha/(1-\alpha)$	$\alpha/(1-\alpha)$	Branching nuclei
**4. Deceleration rate equations**				
**4.1 Phase boundary reaction**				
24	R_1, F_0, P_1	α	$(1-\alpha)^0$	Contracting disk
25	R_2, $F_{1/2}$	$1-(1-\alpha)^{1/2}$	$2\,(1-\alpha)^{1/2}$	Contraction cylinder (cylindrical symmetry)
26	R_3, $F_{2/3}$	$1-(1-\alpha)^{1/3}$	$3\,(1-\alpha)^{2/3}$	Contraction cylinder (spherical symmetry)
**4.2 Based on the diffusion mechanism**				
27	D_1	α^2	$1/(2\alpha)$	One-dimensional diffusion
28	G_7	$[1-(1-\alpha)^{1/2}]^{1/2}$	$4\{(1-\alpha)\,[1-(1-\alpha)^{1/2}]\}^{1/2}$	Two-dimensional diffusion
29	D_2	$\alpha+(1-\alpha)\ln(1-\alpha)$	$[-\ln(1-\alpha)]^{-1}$	Two-dimensional diffusion
30	D_3	$[1-(1-\alpha)^{1/3}]^2$	$3/2(1-\alpha)^{2/3}[1-(1-\alpha)^{1/3}]^{-1}$	Three-dimensional diffusion, spherical symmetry
31	D_4	$1-2/3\alpha-(1-\alpha)^{2/3}$	$3/2[(1-\alpha)^{-1/3}-1]^{-1}$	Three-dimensional diffusion, spherical symmetry
32	D_5	$[(1-\alpha)^{-1/3}-1]^2$	$3/2(1-\alpha)^{4/3}[(1-\alpha)^{-1/3}-1]^{-1}$	Three-dimensional diffusion
33	D_6	$[(1+\alpha)^{-1/3}-1]^2$	$3/2(1+\alpha)^{4/3}[(1+\alpha)^{-1/3}-1]^{-1}$	Three-dimensional diffusion
34	D_7	$1+2/3\alpha-(1+\alpha)^{+2/3}$	$3/2[(1+\alpha)^{-1/3}-1]^{-1}$	Three-dimensional diffusion
35	D_8	$[(1+\alpha)^{-1/3}-1]^2$	$3/2(1+\alpha)^{4/3}[(1+\alpha)^{-1/3}-1]^{-1}$	Three-dimensional diffusion
36	G_8	$[1-(1-\alpha)^{-1/3}]^{1/2}$	$6(1-\alpha)^{2/3}[1-(1-\alpha)^{1/3}]^{1/2}$	Three-dimensional diffusion

Tab. S2: E_a (kJ/mol) and R^2 for the thermal degradation of UiO-66, calculated using the KAS and FWO methods without the iterative procedure

α		Step I			Step II		
		**E_a/KJ/mol**	**R^2**	**A/min^{-1}**	**E_a/KJ/mol**	**R^2**	**A/min^{-1}**
KAS	0.1	93.38	0.9893	-	254.20	0.9984	-
	0.2	104.55	0.9983	-	261.55	0.9999	-
	0.3	126.54	0.9974	-	284.97	0.9985	-
	0.4	107.55	0.9999	-	260.45	0.9999	-
	0.5	113.24	0.9902	-	275.81	0.9999	-
	0.6	96.344	0.9873	-	259.97	0.9972	-
	0.7	107.46	0.9987	-	268.73	0.9904	-
	0.8	128.80	0.9997	-	277.64	0.9983	-
	0.9	131.39	0.9997	-	285.91	0.9982	-

Average		112.14			269.91		
FWO	0.1	106.75	0.9925	-	260.64	0.9986	-
	0.2	117.03	0.9988	-	267.89	0.9999	-
	0.3	137.66	0.9980	-	290.43	0.9987	-
	0.4	119.56	0.9999	-	267.24	0.9999	-
	0.5	124.98	0.9927	-	281.97	0.9991	-
	0.6	108.94	0.99105	-	267.05	0.9976	-
	0.7	119.57	0.9990	-	275.63	0.9918	-
	0.8	139.95	0.9997	-	284.34	0.9985	-
	0.9	142.50	0.9997	-	292.58	0.9984	-
Average		124.10			276.42		

Tab. S3: E_a (kJ/mol) and R^2 for the thermal degradation of UiO-66-W(25%), calculated using the KAS and FWO methods without the iterative procedure

α		Step I			Step II			Step III		
		E_a	R^2	A	E_a	R^2	A	E_a	R^2	A
KAS	0.1	84.84	0.9972	-	256.07	0.9828	-	321.79	0.9915	-
	0.2	95.08	0.9994	-	251.21	0.9800	-	333.95	0.9958	-
	0.3	111.04	0.9990	-	243.14	0.9790	-	326.75	0.9967	-
	0.4	111.13	0.9864	-	244.69	0.9917	-	318.07	0.9974	-
	0.5	112.05	0.9987	-	262.97	0.9965	-	335.69	0.9907	-
	0.6	99.12	0.9997	-	241.25	0.9905	-	322.52	0.9975	-
	0.7	103.66	0.9759	-	238.22	0.9984	-	333.13	0.9999	-
	0.8	107.61	0.9974	-	251.14	0.9984	-	324.52	0.9977	-
	0.9	100.67	0.9993	-	254.47	0.9999	-	330.98	0.9979	-
Average		102.80			249.24			327.49		
FWO	0.1	98.99	0.9980	-	261.89	0.9851	-	328.32	0.9926	-
	0.2	108.39	0.9995	-	257.56	0.9828	-	340.39	0.9964	-
	0.3	123.22	0.9992	-	250.24	0.9821	-	333.86	0.9972	-
	0.4	123.16	0.9899	-	252.00	0.9929	-	325.80	0.9977	-
	0.5	123.90	0.9991	-	269.57	0.9970	-	342.82	0.9919	-
	0.6	111.54	0.9998	-	249.06	0.9921	-	330.47	0.9979	-
	0.7	115.92	0.9825	-	246.52	0.9987	-	340.81	0.9999	-
	0.8	119.82	0.9981	-	259.27	0.9986	-	332.88	0.9980	-
	0.9	113.36	0.99957	-	262.65	0.9999	-	339.38	0.9982	-
Average		115.37		-	256.53		-	334.97		-

Tab. S4: E_a (kJ/mol) and R^2 for the thermal degradation of UiO-66-W(60%), calculated using the KAS and FWO methods without the iterative procedure

α		Step I			Step II			Step III		
		E_a	R^2	A	E_a	R^2	A	E_a	R^2	A
KAS	0.1	49.84	0.9965	-	228.67	0.9957	-	359.83	0.9998	-
	0.2	56.67	0.9907	-	234.65	0.9969	-	352.54	0.9961	-
	0.3	50.63	0.9996	-	238.58	0.9883	-	349.68	0.9978	-
	0.4	56.46	0.9985	-	224.29	0.9963	-	356.00	0.9957	-
	0.5	58.97	0.9993	-	238.93	0.9954	-	353.12	0.9955	-
	0.6	81.53	0.9985	-	237.51	0.9999	-	346.29	0.9993	-
	0.7	92.78	0.9946	-	233.26	0.9991	-	345.38	0.9988	-

	0.8	106.3	0.9998	-	221.52	0.9999	-	358.84	0.9951	-
	0.9	91.27	0.9929	-	228.60	0.9968	-	345.83	0.9999	-
Average		71.61			231.78			351.95		
FWO	0.1	66.02	0.9980	-	236.01	0.9963	-	364.76	0.9998	-
	0.2	71.85	0.9945	-	241.95	0.9974	-	358.07	0.9966	-
	0.3	65.80	0.9997	-	245.89	0.9900	-	355.69	0.9981	-
	0.4	71.11	0.9991	-	232.50	0.9969	-	361.95	0.9962	-
	0.5	73.39	0.9996	-	246.74	0.9961	-	359.39	0.9961	-
	0.6	94.82	0.9990	-	245.75	0.9999	-	353.15	0.9994	-
	0.7	105.51	0.9963	-	242.02	0.9993	-	352.48	0.9990	-
	0.8	118.49	0.9999	-	231.08	0.9999	-	365.48	0.9956	-
	0.9	104.34	0.9950	-	238.01	0.9973	-	353.28	0.9999	-
Average		85.71		-	239.99		-	358.25		-

Tab. S5: E_a (KJ/mol), R^2 and pre-exponential factor A (min^{-1}) calculated using FWO-Ir and KAS-Ir methods for the thermal degradation of UiO-66

α		Step I			Step II		
		E_a	R^2	A	E_a	R^2	A
KAS	0.1	102.20	0.9911	4.26E8	263.52	0.9970	3.77E14
	0.2	113.21	0.9986	1.83E10	271.01	0.9999	1.20E15
	0.3	135.05	0.9977	1.40E12	294.54	0.9972	2.44E16
	0.4	116.17	0.9999	9.91E9	270.14	0.9999	6.07E14
	0.5	121.87	0.9915	4.03E10	285.54	0.9981	4.95E15
	0.6	105.06	0.9893	1.29E9	269.81	0.9949	3.95E14
	0.7	116.19	0.9989	1.57E10	278.71	0.9822	6.82E14
	0.8	137.53	0.9997	1.41E12	287.72	0.9969	1.16E15
	0.9	140.17	0.9997	3.51E12	296.19	0.9966	1.12E15
Average		120.83		7.13E11	279.69		3.88E15
FWO	0.1	102.26	0.9911	2.85E9	263.58	0.9970	4.31E14
	0.2	113.25	0.9986	9.13E10	271.07	0.9999	1.35E15
	0.3	135.11	0.9977	4.58E12	294.60	0.9972	2.43E16
	0.4	116.22	0.9999	4.31E10	270.20	0.9999	7.32E14
	0.5	121.91	0.9915	1.59E11	285.60	0.9981	5.46E15
	0.6	105.11	0.9893	6.97E9	269.87	0.9949	4.97E14
	0.7	116.23	0.9989	7.02E10	278.75	0.9822	8.35E14
	0.8	137.57	0.9997	4.57E12	287.78	0.9969	1.38E15
	0.9	140.21	0.9997	1.11E13	296.25	0.9966	1.32E15
Average		120.87		2.29E12	279.74		4.04E15

Tab. S6: E_a (KJ/mol), R^2 and pre-exponential factor A (min^{-1}) calculated using FWO-Ir and KAS-Ir methods for the thermal degradation of UiO-66-W(25%)

α		Step I			Step II			Step III		
		E_a	R^2	A	E_a	R^2	A	E_a	R^2	A
	0.1	93.83	0.9976	1.10E8	265.07	0.9679	1.24E16	332.91	0.9841	2.73E13
	0.2	103.89	0.9995	6.83E9	260.39	0.9628	9.94E15	345.33	0.9922	1.34E14

KAS	0.3	119.69	0.9991	3.86E11	252.55	0.9612	1.95E15	338.31	0.9939	6.91E13
	0.4	119.72	0.9883	5.72E11	254.27	0.9847	2.04E15	329.75	0.9951	3.50E13
	**0.5**	120.62	0.9989	4.10E11	272.62	0.9936	3.52E16	347.49	0.9826	2.68E14
	0.6	107.76	0.9997	1.18E10	251.03	0.9825	1.91E15	334.43	0.9955	7.66E13
	0.7	112.37	0.9795	1.09E10	248.19	0.9972	7.96E14	345.16	0.9999	2.57E14
	0.8	116.42	0.9978	1.59E10	261.35	0.9971	2.56E15	336.71	0.9958	1.26E14
	0.9	109.59	0.9994	5.02E9	264.80	0.9999	6.04E15	343.35	0.9962	3.08E14
Average		111.54		1.57E11	258.92		8.10E15	339.27		1.44E14
FWO	0.1	93.89	0.9976	9.65E8	265.13	0.9679	1.29E16	332.97	0.9841	3.31E13
	0.2	103.95	0.9995	4.46E10	260.45	0.9629	1.12E16	345.39	0.9922	1.53E14
	0.3	119.75	0.9991	1.71E12	252.61	0.9612	2.48E15	338.37	0.9939	8.51E13
	0.4	119.77	0.9883	2.46E12	254.32	0.9847	2.67E15	329.81	0.9951	4.64E13
	**0.5**	120.67	0.9989	1.68E12	272.67	0.9936	4.13E16	347.55	0.9826	3.29E14
	0.6	107.80	0.9997	5.99E10	251.08	0.9825	2.70E15	334.49	0.9955	1.03E14
	0.7	112.41	0.9795	5.16E10	248.24	0.9972	1.21E15	345.22	0.9999	3.36E14
	0.8	116.46	0.9978	7.23E10	261.40	0.9971	3.77E15	336.77	0.9958	1.78E14
	0.9	109.62	0.9994	2.64E10	264.85	0.9999	8.90E15	343.41	0.9962	4.34E14
Average		111.59		6.80E11	258.97		9.69E15	339.33		1.88E14

Tab. S7: E_a (KJ/mol), R^2 and pre-exponential factor A (min^{-1}) calculated using FWO-Ir and KAS-Ir methods for the thermal degradation of UiO-66-W(60%)

α		Step I			Step II			Step III		
		E_a	R^2	A	E_a	R^2	A	E_a	R^2	A
KAS	0.1	59.18	0.9974	8072.88	237.84	0.9921	1.25E15	371.03	0.9996	9.79E14
	0.2	65.70	0.9929	169287.26	243.96	0.9943	2.58E15	363.89	0.9928	9.54E14
	0.3	59.58	0.9997	56306.05	247.99	0.9784	3.45E15	361.22	0.9959	6.61E14
	0.4	65.30	0.9989	307866.59	233.85	0.9932	2.45E14	367.67	0.9921	1.35E15
	**0.5**	67.77	0.9994	635869.23	248.64	0.9915	9.17E14	364.89	0.9916	1.08E15
	0.6	90.26	0.9988	1.25E8	247.43	0.9998	2.66E14	358.21	0.9987	4.57E14
	0.7	101.57	0.9955	1.20E9	243.36	0.9985	6.59E13	357.41	0.9979	4.12E14
	0.8	115.05	0.9998	2.41E10	231.76	0.9999	7.88E12	370.95	0.9906	1.75E15
	0.9	100.17	0.9941	1.14E9	238.95	0.9942	1.50E13	358.06	0.9998	4.82E14
Average		80.51		2.96E9	241.53		9.78E14	363.70		9.03E14
FWO	0.1	59.22	0.9974	195056.45	237.88	0.9924	1.65E15	371.09	0.9996	9.39E14
	0.2	65.74	0.9929	2.74E6	244.01	0.9943	3.39E15	363.95	0.9928	9.79E14
	0.3	59.61	0.9997	1.79E6	248.04	0.9784	4.53E15	361.28	0.9959	7.16E14
	0.4	65.33	0.9989	4.37E6	233.91	0.9932	3.74E14	367.73	0.9922	1.45E15
	**0.5**	67.80	0.9998	8.18E6	248.71	0.9915	1.30E15	364.95	0.9916	1.20E15
	0.6	90.29	0.9988	9.05E8	247.48	0.9998	4.01E14	358.27	0.9987	5.43E14
	0.7	101.58	0.9955	6.94E9	243.41	0.9985	1.07E14	357.47	0.9979	5.03E14
	0.8	115.09	0.9998	1.10E11	231.82	0.9999	1.46E13	371.01	0.9906	2.02E15
	0.9	100.20	0.9941	7.11E9	239.01	0.9942	2.70E13	358.11	0.9998	6.10E14
Average		80.54		1.39E10	241.58		1.31E15	363.76		9.98E14

Tab. S8: The α-β value for the plots of lng(α) versus lnβ for the thermal degradation of UiO-66, UiO-66-W(25%) and UiO-66-W(60%)

β/(°C min^{-1})	Step and α values at selected temperatures/ °C

	UiO-66		UiO-66-W(25%)			UiO-66-W(60%)		
	I	II	I	II	III	I	II	III
	278.46	**550.83**	247.15	**543.41**	859.57	231.73	540.42	851.71
10	**0.5269**	**0.5109**	0.5368	**0.4739**	0.6119	0.501	**0.319**	0.5807
30	**0.4661**	**0.2709**	0.441	**0.3624**	0.4325	0.4131	**0.1734**	0.3682
50	**0.3349**	**0.1885**	0.3272	**0.3031**	0.3757	0.3384	**0.1001**	0.2905

Tab. S9: Slope, correlation coefficient and the most probable mechanism function g(α) for the thermal degradation of UiO-66, UiO-66-W(25%) and UiO-66-W(60%)

Sample	Step	Symbol	g(α)	Slope	R^2
UiO-66	I	G_5	$[-\ln(1-\alpha)]^3$	-1.0282	0.8143
		G_6	$[-\ln(1-\alpha)]^4$	-1.3710	0.8143
	II	F_2	$(1-\alpha)^{-1}-1$	-0.9351	0.9999
		F_3	$(1-\alpha)^{-2}-1$	-1.1335	0.9992
		$P_{3/2}$	$(\alpha)^{3/2}$	-0.9192	0.9973
		D_7	$1 + 2/3\alpha - (1+\alpha)^{+2/3}$	-1.1532	0.9966
		D_8	[(1+a)-1/3 - 1]2	-1.0184	0.9949
		F_2	$(1-\alpha)^{-1}-1$	-0.9351	0.9999
UiO-66-W(25%)	I	G_5	$[-\ln(1-\alpha)]^3$	-1.1619	0.9140
		G_6	$[-\ln(1-\alpha)]^4$	-1.5492	0.9140
	II	G_5	$[-\ln(1-\alpha)]^3$	-1.0570	0.9947
		G_6	$[-\ln(1-\alpha)]^4$	-1.4094	0.9947
	III	F_3	$(1-\alpha)^{-2}-1$	-0.8122	0.9913
		G_4	$[-\ln(1-\alpha)]^2$	-0.8776	0.9966
		G_5	$[-\ln(1-\alpha)]^3$	-1.3165	0.9966
		D_5	[(1 - a)-1/3 -1]2	-0.9828	0.9957
UiO-66-W(60%)	I	G_5	$[-\ln(1-\alpha)]^3$	-0.9312	0.9622
		G_6	$[-\ln(1-\alpha)]^4$	-1.2416	0.9622
	II	F_3	$(1-\alpha)^{-2}-1$	-0.9652	0.9848
		F_4	$(1-\alpha)^{-3}-1$	-1.0699	0.9884
		$P_{3/2}$	$(\alpha)^{3/2}$	-1.0407	0.9687
		G_4	$[-\ln(1-\alpha)]^2$	-1.5541	0.9751
		D_8	[(1+a)-1/3 - 1]2	-1.2355	0.9626
	III	F_3	$(1-\alpha)^{-2}-1$	-0.9788	0.9974
		G_4	$[-\ln(1-\alpha)]^2$	-1.1558	0.9999
		D_3	[1 - (1 - a)1/3]2	-1.0482	0.9999
		D_4	$1-2/3\alpha-(1-\alpha)^{2/3}$	-0.9792	0.9998

Tab. S10: Thermodynamic parameters ($\Delta S^{\neq}$ /J/molK, $\Delta H^{\neq}$ /kJ/mol and $\Delta G^{\neq}$ /kJ/mol) for the thermal decomposition of UiO-66

**α**	**Step 1**						**Step 2**					
	**KAS-Ir**			**FWO-Ir**			**KAS-Ir**			**FWO-Ir**		
	$\Delta S^{\neq}$	$\Delta H^{\neq}$	$\Delta G^{\neq}$	$\Delta S^{\neq}$	$\Delta H^{\neq}$	$\Delta G^{\neq}$	$\Delta S^{\neq}$	$\Delta H^{\neq}$	$\Delta G^{\neq}$	$\Delta S^{\neq}$	$\Delta H^{\neq}$	$\Delta G^{\neq}$
0.1	-93.16	97.61	148.98	-77.32	97.67	140.32	17.38	256.67	242.35	18.49	256.73	241.49
0.2	-61.87	108.69	142.74	-48.51	108.68	135.43	27.00	264.16	241.91	28.01	264.22	241.13
0.3	-25.76	130.47	144.68	-15.95	130.52	139.33	52.04	287.69	244.80	52.01	287.75	244.89
0.4	-66.97	111.59	148.53	-54.75	111.63	141.83	21.32	263.29	245.72	22.89	263.35	244.48
0.5	-55.31	117.28	147.80	-43.85	117.33	141.52	38.78	278.69	246.73	39.59	278.75	246.12
0.6	-83.88	100.48	146.75	-69.90	100.51	139.07	17.76	262.96	248.32	19.67	263.02	246.80
0.7	-63.10	111.60	146.41	-50.69	111.64	139.61	22.29	271.85	253.47	23.98	271.90	252.14
0.8	-25.73	132.94	147.14	-15.98	132.99	141.80	26.76	280.87	258.82	28.20	280.93	257.69
0.9	-18.17	135.59	145.61	-8.58	135.63	140.36	26.42	289.34	267.56	27.81	289.40	266.48
**Average**	**-54.88**	**116.24**	**146.52**	**-42.84**	**116.29**	**139.92**	**27.75**	**272.83**	**249.96**	**28.96**	**272.89**	**249.03**

Tab. S11: Thermodynamic parameters ($\Delta S^{\neq}$ /J/molK, $\Delta H^{\neq}$ /kJ/mol and $\Delta G^{\neq}$ /kJ/mol) for the thermal degradation of UiO-66-W(25%)

**α**	**Step 1**						**Step 2**						**Step 3**					
	**KAS-Ir**			**FWO-Ir**			**KAS-Ir**			**FWO-Ir**			**KAS-Ir**			**FWO-Ir**		
	$\Delta S^{\neq}$	$\Delta H^{\neq}$	$\Delta G^{\neq}$	$\Delta S^{\neq}$	$\Delta H^{\neq}$	$\Delta G^{\neq}$	$\Delta S^{\neq}$	$\Delta H^{\neq}$	$\Delta G^{\neq}$	$\Delta S^{\neq}$	$\Delta H^{\neq}$	$\Delta G^{\neq}$	$\Delta S^{\neq}$	$\Delta H^{\neq}$	$\Delta G^{\neq}$	$\Delta S^{\neq}$	$\Delta H^{\neq}$	$\Delta G^{\neq}$
0.1	-103.91	89.51	143.57	-85.85	89.57	134.23	46.54	258.28	220.27	46.83	258.34	220.10	-7.09	323.49	331.53	-5.48	307.21	313.42
0.2	-69.58	99.57	135.77	-53.98	99.63	127.72	44.65	253.60	217.14	45.64	253.66	216.39	6.17	335.91	328.91	7.25	335.97	327.76
0.3	-36.04	115.33	134.11	-23.62	115.43	127.72	31.13	245.76	220.34	33.11	245.82	218.77	0.62	328.89	328.19	2.35	328.95	326.28
0.4	-32.77	115.39	132.44	-20.61	115.45	126.17	31.48	247.48	221.77	33.72	247.53	219.99	-5.02	320.33	326.02	-2.69	320.39	323.42
0.5	-35.54	116.30	134.79	-23.81	116.35	128.74	55.16	265.83	220.78	56.49	265.88	219.75	11.88	338.07	324.61	13.59	338.13	322.73
0.6	-65.02	103.44	137.26	-51.53	103.48	130.29	30.93	244.24	218.98	33.82	244.29	216.67	1.47	325.02	323.34	3.99	325.07	320.54
0.7	-65.67	108.05	142.22	-52.76	108.09	135.54	23.66	241.40	222.08	27.16	241.45	219.27	11.54	335.74	322.66	13.76	335.80	320.21
0.8	-62.54	112.10	144.64	-49.97	112.13	138.13	33.39	254.56	227.29	36.58	254.61	224.73	5.62	327.29	320.93	8.48	327.35	317.74
0.9	-72.15	105.26	142.80	-58.34	105.30	135.65	40.51	258.01	224.92	43.73	258.06	222.34	13.04	333.94	319.16	15.89	333.99	315.98
**Av***	**-60.36**	**107.22**	**138.62**	**-46.72**	**107.27**	**131.58**	**37.49**	**252.13**	**221.51**	**39.68**	**252.18**	**219.78**	**4.25**	**329.85**	**325.04**	**6.35**	**328.10**	**320.90**

Av : Average*

Tab. S12: Thermodynamic parameters ($\Delta S^{\neq}$ /J/molK, $\Delta H^{\neq}$ /kJ/mol and $\Delta G^{\neq}$ /kJ/mol) for the thermal degradation of UiO-66-W(60%)

**α**	**Step 1**						**Step 2**						**Step 3**					
	**KAS-Ir**			**FWO-Ir**			**KAS-Ir**			**FWO-Ir**			**KAS-Ir**			**FWO-Ir**		
	$\Delta S^{\neq}$	$\Delta H^{\neq}$	$\Delta G^{\neq}$	$\Delta S^{\neq}$	$\Delta H^{\neq}$	$\Delta G^{\neq}$	$\Delta S^{\neq}$	$\Delta H^{\neq}$	$\Delta G^{\neq}$	$\Delta S^{\neq}$	$\Delta H^{\neq}$	$\Delta G^{\neq}$	$\Delta S^{\neq}$	$\Delta H^{\neq}$	$\Delta G^{\neq}$	$\Delta S^{\neq}$	$\Delta H^{\neq}$	$\Delta G^{\neq}$
0.1	-182.81	54.98	147.28	-156.33	55.03	133.96	27.47	231.07	208.72	29.79	231.12	206.88	22.71	361.68	336.13	22.36	361.74	336.58
0.2	-157.51	61.51	141.02	-134.34	61.54	129.37	33.47	237.19	209.96	35.73	237.25	208.17	22.50	354.54	329.22	22.71	354.60	329.05
0.3	-166.66	55.38	139.52	-142.57	55.41	127.39	35.88	241.23	212.04	38.16	241.28	210.23	19.45	351.87	329.98	20.11	351.93	329.30
0.4	-152.53	61.11	138.11	-130.47	61.13	127.06	13.90	227.08	215.77	17.41	227.14	212.98	25.38	358.31	329.75	25.99	358.37	329.13
0.5	-146.51	63.57	137.54	-125.26	63.61	126.85	24.86	241.88	221.64	27.77	241.94	219.34	23.55	355.53	329.04	24.46	355.59	328.08
0.6	-102.56	86.06	137.84	-86.13	86.09	129.58	14.57	240.67	228.81	18.00	240.71	226.06	16.38	348.86	330.43	17.81	348.92	328.87
0.7	-83.76	97.32	139.61	-69.19	97.36	132.29	2.97	236.60	234.17	7.06	236.65	230.90	15.53	348.06	330.58	17.19	348.11	328.77
0.8	-58.82	110.8	140.56	-46.21	110.85	134.23	-14.67	225.01	236.94	-9.52	225.06	232.79	27.55	361.60	330.60	28.76	361.66	329.30
0.9	-84.15	95.97	138.45	-68.99	96.01	130.83	-9.29	232.19	239.75	-4.41	232.23	235.83	16.82	348.71	329.78	18.78	348.76	327.63
**Av***	**-126.14**	**76.31**	**139.99**	**-106.61**	**76.34**	**130.17**	**14.35**	**234.77**	**223.09**	**17.78**	**234.82**	**220.35**	**21.11**	**354.35**	**330.61**	**22.02**	**354.41**	**329.63**

Av : Average*

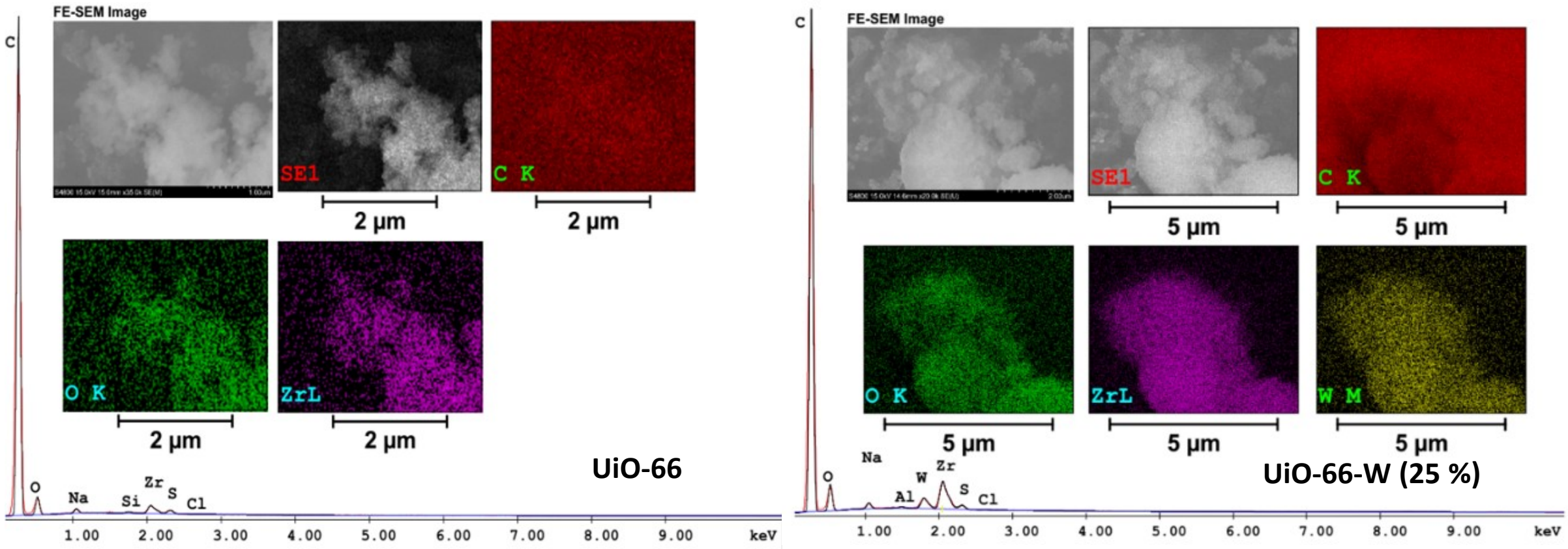

Fig. S1. EDX and EDX elemental distribution mapping

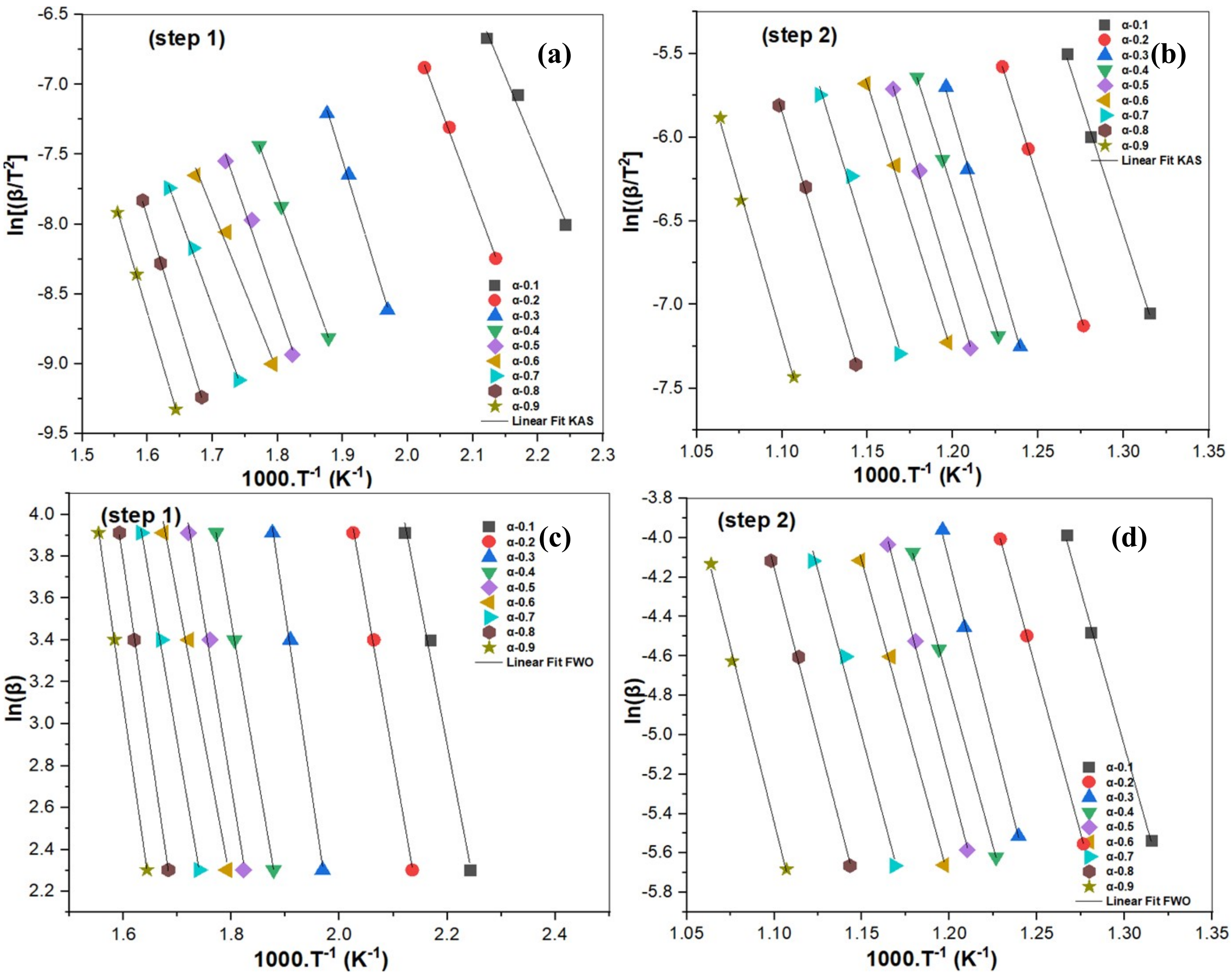

Fig. S2. KAS (a and b) and FWO methods (c and d) for steps 1 and 2, respectively, for UiO-66

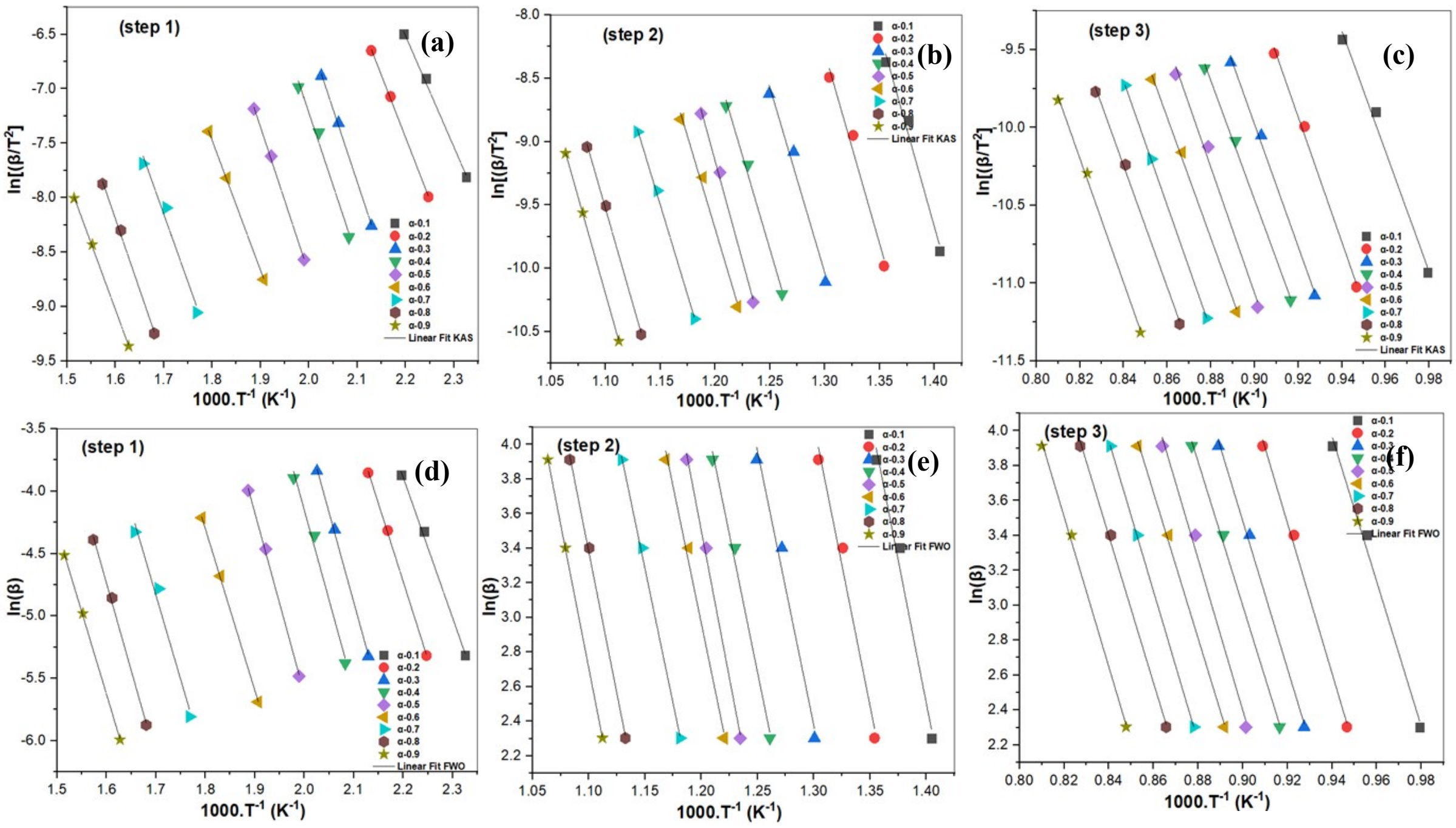

Fig. S3. KAS (a, b and c) and FWO methods (d, e and f) for steps 1 and 2, respectively, for UiO-66-W (25 %)

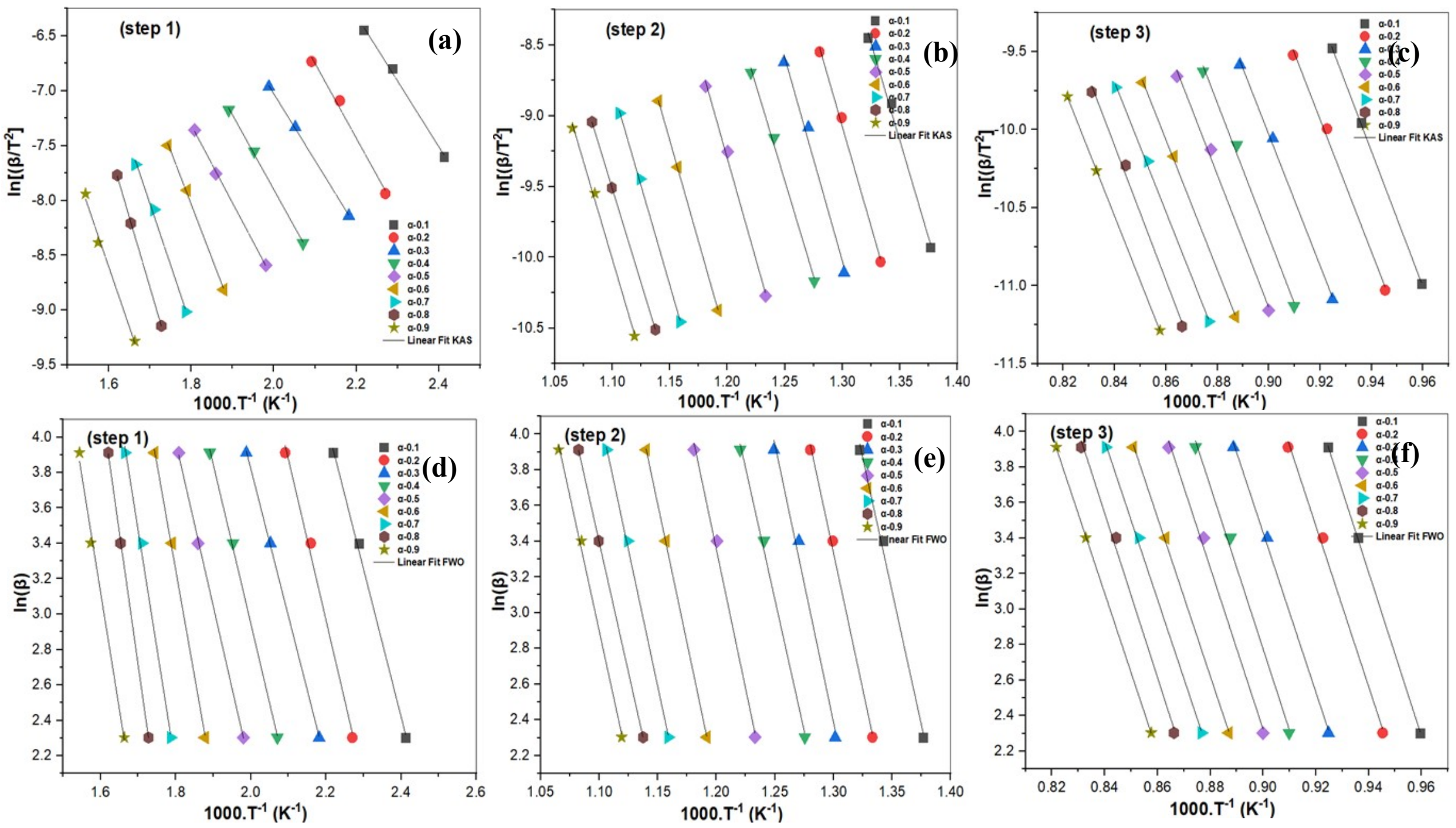

Fig. S4. KAS (a, b and c) and FWO methods (d, e and f) for steps 1 and 2, respectively, for UiO-66-W (60 %)

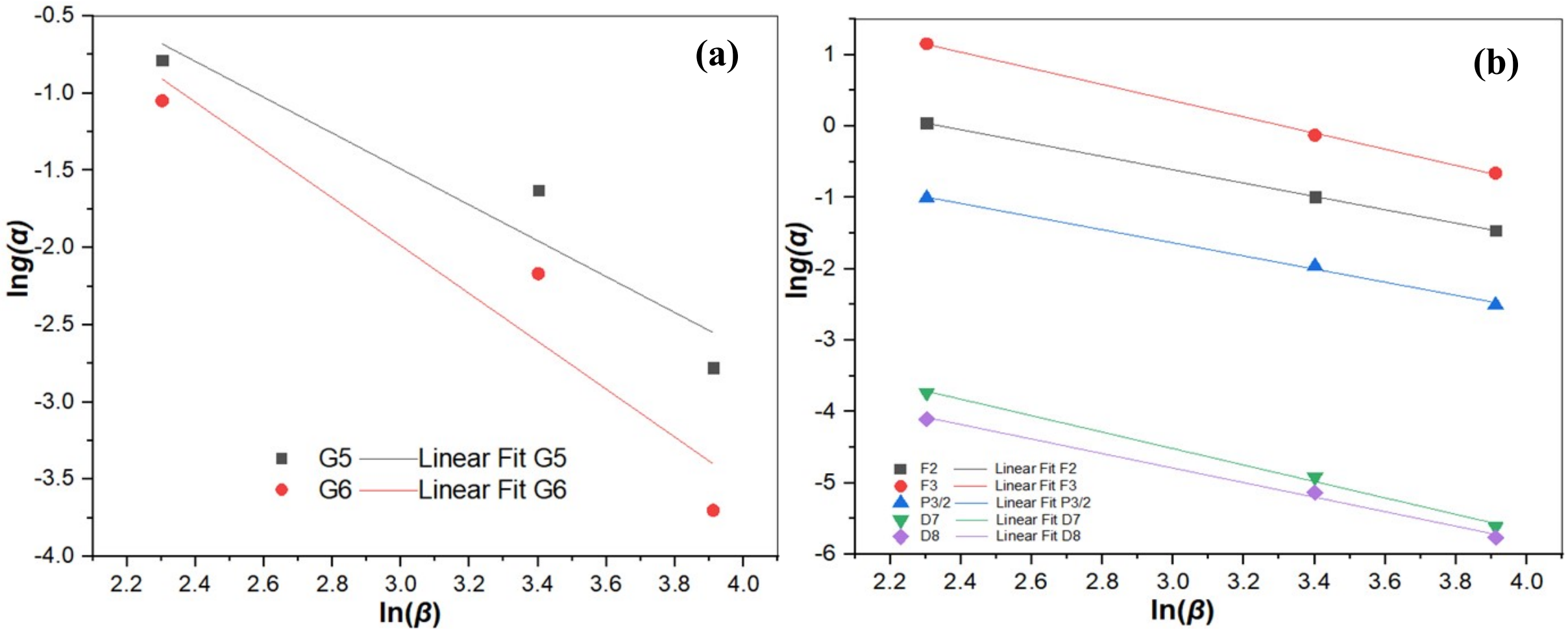

Fig. S5. Plot of $lng(\alpha)$ versus $\ln(\beta)$ for UiO-66 (a and b) for steps 1 and 2, respectively, of the thermal degradation

References

[1] H. Jiang, J. Wang, S. Wu, B. Wang, et Z. Wang, « Pyrolysis kinetics of phenol – formaldehyde resin by non-isothermal thermogravimetry », *Carbon*, vol. 48, nº 2, p. 352-358, 2009, doi: 10.1016/j.carbon.2009.09.036.

[2] C. Sronsri, P. Noisong, et C. Danvirutai, « Isoconversional kinetic, mechanism and thermodynamic studies of the thermal decomposition of NH4Co0.8Zn0.1Mn0.1PO4·H2O », *J. Therm. Anal. Calorim.*, vol. 120, nº 3, p. 1689-1701, juin 2015, doi: 10.1007/s10973-015-4471-x.
